# DDI-4 induces a temperature-sensitive exocytotic remodeling during *C. elegans* spermiogenesis *via nsun-2*–dependent sphingosine signaling

**DOI:** 10.1371/journal.pgen.1012275

**Published:** 2026-08-27

**Authors:** Yoshihiro Shimada, Riona Shiraki, Chihiro Ogawa, Nana Kanazawa-Takino, Yukiko Karuo, Kentaro Kawai, Masaharu Hashimoto, Ayaka Yoshida, Arata Honda, Jun Adachi, Jun-Dal Kim, Akiyoshi Fukamizu, Masaaki Omote, Hitoshi Nishimura

**Affiliations:** 1 Department of Life Science, Faculty of Science and Engineering, Setsunan University, Neyagawa, Osaka, Japan; 2 Faculty of Pharmaceutical Sciences, Setsunan University, Hirakata, Osaka, Japan; 3 Center for Development of Advanced Medical Technology, Jichi Medical University School of Medicine, Shimotsuke, Tochigi, Japan; 4 Laboratory of Proteomics for Drug Discovery, Center for Drug Design Research, National Institutes of Biomedical Innovation, Health and Nutrition, Ibaraki, Osaka, Japan; 5 Division of Complex Biosystem Research, Department of Research and Development, Institute of Natural Medicine, University of Toyama, Toyama, Toyama, Japan; 6 Life Science Center for Survival Dynamics, Tsukuba Advanced Research Alliance, University of Tsukuba, Tsukuba, Ibaraki, Japan; Queens College and the Graduate Center, CUNY, UNITED STATES OF AMERICA

## Abstract

Exocytotic sperm remodeling is essential for fertilization, yet whether conserved molecular mechanisms underlie this process across species remains unclear. In *Caenorhabditis elegans*, membranous organelle fusion (MOF), an exocytotic event cytologically analogous to the mammalian acrosome reaction (ASR), occurs during spermiogenesis and likely contributes to the acquisition of fertilization competence. Here, we identify DDI-4, a benzylamine analog as a small-molecule inducer of both MOF and ASR, revealing a shared sensitivity between these evolutionarily distant systems. DDI-4–induced MOF was selectively impaired in spermatids from males raised at elevated temperatures, whereas conventional protease–induced MOF remained unaffected. Through forward genetics, we isolated the *nyg20* mutant, which was specifically defective in DDI-4 responsiveness. Complementation and genetic analyses suggested that the *nyg20* phenotype is associated with impaired function of *nsun-2*, encoding a tRNA methyltransferase, despite the absence of detectable mutations in the *nsun-2* coding, intronic, and flanking regions. Consistent with this, an *nsun-2* deletion mutant exhibited defects in temperature-sensitive MOF and meiosis. *In silico* docking analysis further implicated sphingosine kinases (SPHKs) as candidate targets of DDI-4; indeed, loss of *sphk-1* phenocopied the *nsun-2* mutant. Moreover, we tested sphingosine (SPH) and its analog FTY720 as MOF activators, using mutants lacking *sphk-1* or *spin-4*, which encodes a transporter of phosphorylated SPH. Intriguingly, DDI-4 and SPH activated MOF in an SPHK-1–dependent but SPIN-4–independent manner, whereas FTY720 required both SPHK-1 and SPIN-4 for MOF activation. Because DDI-4 lacks hydroxyl groups that SPHKs typically phosphorylate, these findings suggest that SPHK-1 may function not only as a kinase but also as a scaffold for downstream signaling. Together, our results reveal a temperature-sensitive, NSUN-2–dependent SPH-mediated signaling axis that regulates organelle exocytosis during spermiogenesis and suggest an evolutionarily conserved mechanism underlying fertilization competence.

## Author summary

Fertilization requires exocytotic sperm remodeling, but whether similar molecular mechanisms govern this process across species remains poorly understood. In this study, we used the nematode *Caenorhabditis elegans* to investigate how the exocytotic event provisionally termed membranous organelle fusion (MOF), which likely contributes to the acquisition of fertilization competence, is regulated. We identified a small molecule, DDI-4, which can trigger exocytotic sperm remodeling not only in worms but also in mice, suggesting a shared underlying mechanism. We found that this response is temperature-sensitive and depends on the *nsun-2* gene, which is involved in translational regulation by RNA modification. When this gene is disrupted, sperm fail to respond properly under elevated rearing temperatures. Our results also suggest a role for lipid signaling molecules, particularly sphingosine and its associated enzyme SPHK-1, in regulating this process. Interestingly, SPHK-1 may function not only as an enzyme but also as a structural platform that helps organize signaling events. Together, our findings reveal a previously unrecognized pathway that links temperature sensitivity, RNA regulation, and lipid signaling to MOF. This work provides new insight into how fertilization is controlled and suggests that common mechanisms may be shared across evolutionarily distant species.

## Introduction

Exocytotic sperm remodeling is a fundamental cellular transition that likely contributes to the acquisition of fertilization competence across diverse animal species, including mammals [1]. Despite its central role in sexual reproduction, the regulatory principles governing the exocytotic event and the extent to which these mechanisms are conserved through evolution remain poorly understood.

Generally, exocytotic sperm remodeling includes changes in membrane organization, ion homeostasis, and organelle-specific exocytosis [1]. These processes are hallmarks of regulated secretion in many cell types, yet in spermatozoa, they are executed within a highly specialized transcriptionally silent cellular context. Understanding how such diverse cellular events are coordinated to drive fertilization competence, therefore, offers a unique opportunity to uncover general principles of cellular regulation within a developmentally and evolutionarily constrained system.

The nematode *Caenorhabditis elegans* provides a powerful genetic model to dissect the mechanisms of exocytotic sperm remodeling. In this organism (S1A Fig), the sperm event occurs during spermiogenesis, transforming post-meiotic round spermatids into motile spermatozoa [2–7]. This transition involves fusion of membranous organelles (MOs) with the spermatid plasma membrane (PM), an exocytotic reaction hereafter referred to as membranous organelle fusion (MOF). Concomitantly, a subset of SPE-9 class proteins essential for fertilization redistributes from the MO membrane to the entire sperm surface [7,8], thereby enabling sperm–oocyte binding and fusion.

Genetic and pharmacological studies have shown that *C. elegans* spermiogenesis is triggered by an external cue, provisionally termed the spermatid-activating factor (SAF), which likely activates sperm through either SPE-8 class–dependent or –independent signaling pathway [2,3,7]. *In vitro*, these pathways can be selectively stimulated by the bacterial protease mixture Pronase (Pron) [9] or by Proteinase K (ProK) [10], respectively. Despite the detailed characterization of these activation routes, how intracellular signaling pathways are coupled to organelle-specific exocytosis during MOF remains unclear.

In mammals (S1B Fig), the acrosome reaction (ASR) is an exocytotic sperm event that is required for successful fertilization [1,7], during which acrosomal contents are released and the fusogenic protein IZUMO1 relocates to the equatorial segment of the sperm head [11], the site of gamete fusion [12]. Given the striking cytological parallels between MOF and ASR, these processes have long been suspected to share conserved regulatory logic, yet direct experimental evidence supporting this idea has been limited.

*C. elegans* offers a unique experimental advantage for addressing this question, as MOF can be robustly induced *in vitro* using a simple, chemically defined medium. Using this system, previous chemical screens identified small molecules that trigger both MOF and ASR [13], suggesting the existence of conserved pathways underlying exocytotic sperm remodeling. However, the molecular mechanisms by which these compounds act remain unresolved.

Here, we identify a benzylamine-containing compound, DDI-4, that induces temperature-sensitive MOF in *C. elegans* spermatids, whereas Pron- or ProK-triggered MOF was temperature-insensitive. We show that DDI-4–induced MOF requires the tRNA methyltransferase NSUN-2 and the sphingosine kinase SPHK-1, linking translational control and sphingolipid signaling to exocytotic sperm remodeling. Notably, DDI-4 also triggers the ASR and IZUMO1 translocation in mouse spermatozoa, suggesting that elucidation of the DDI-4–responsive pathway in *C. elegans* provides insight into conserved mechanisms regulating mammalian exocytotic sperm remodeling.

## Results

### DDI-4, but neither Pron nor ProK, triggers temperature-sensitive MOF in *C. elegans* spermatids

In a recent study, we screened the Core Library provided by the Drug Discovery Initiative (DDI; University of Tokyo) using an *in vitro C. elegans* spermiogenesis assay [13]. This screening identified one positive compound, DDI-4, which exhibited higher activity than the quinolinol analog DDI-6, previously reported as a dual activator of MOF and ASR [13]. Thus, we further examined how DDI-4 triggers MOF in *C. elegans* spermatids. Although *C. elegans* spermiogenesis is characterized by pseudopod extension from spermatids accompanied by exocytotic remodeling, MOF was assessed exclusively based on FM1-43 staining rather than sperm morphology.

**Fig 1:**
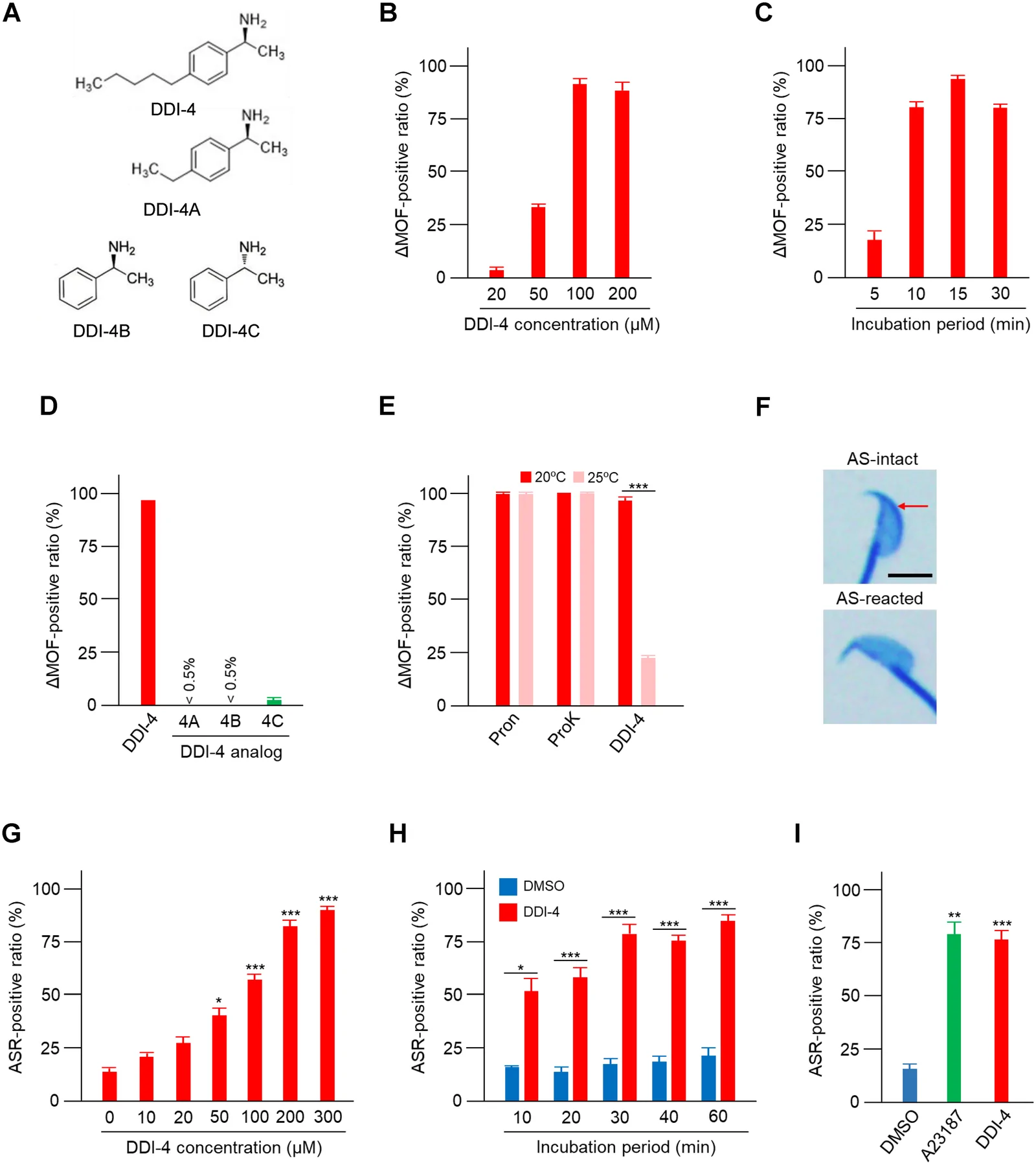
DDI-4 is a dual activator of nematode MOF and mouse ASR. **(A)** Chemical structures of DDI-4 and its analogs. **(B)** Concentration-dependent effect of DDI-4 on MOF. ΔMOF-positive ratios were calculated by subtracting the baseline value obtained with 0.5% dimethyl sulfoxide (DMSO) from those obtained with each DDI-4 concentration in 0.5% DMSO. Net ratios are shown as histograms with the mean ± standard error of the mean (SEM; *n* = 3). **(C)** Incubation period–dependent effect of DDI-4 on MOF. The nematode MOF assay was performed using 0.5% DMSO or 100 μM DDI-4 as SAFs. At each time point, ΔMOF-positive ratios were calculated as described in panel (B) and are shown as histograms indicating the mean ± SEM (*n* = 3). **(D)** Comparison of DDI-4 and its analogs (DDI-4A, DDI-4B, and DDI-4C) in MOF-inducing activity. The nematode MOF assay was performed using 0.5% DMSO, 100 μM DDI-4, or 100 μM DDI-4 analogs. ΔMOF-positive ratios were calculated as described in panel (B) and are shown as histograms indicating the mean ± SEM (*n* = 3). **(E)** Temperature-sensitive MOF triggered by DDI-4. The nematode MOF assay was performed using 1x Sperm Medium (1x SM), 200 μg/mL Pronase (Pron), 200 μg/mL Proteinase K (ProK), 0.5% DMSO, or 100 μM DDI-4. 1 × SM was used as the solvent control for Pron and ProK, and DMSO was used as the solvent control for DDI-4. At each rearing temperature, based on the micrographs shown in S2 Fig, ΔMOF-positive ratios were calculated as described in panel (B) and are shown as histograms indicating the mean ± SEM (*n* = 3). *P*-values for differences in DDI-4–triggered ΔMOF-positive ratios between spermatids raised at 20°C and 25°C are indicated by asterisks. ***, *P* < 0.005. **(F)** Images of acrosome (AS)-intact and AS-reacted mouse spermatozoa after staining with Coomassie Brilliant Blue G-250. A red arrow indicates the AS region. Scale bar = 10 µm. **(G)** Concentration-dependent effect of DDI-4 on ASR. Data are shown as histograms indicating the mean ± SEM (*n* = 3). *, *P* < 0.05; ***, *P* < 0.005. **(H)** Incubation period–dependent effect of DDI-4 on ASR. The mouse ASR assay was performed using 0.1% DMSO or 200 μM DDI-4 as ASR inducers. *, *P* < 0.05; ***, *P* < 0.005. **(I)** Comparison of A23187 and DDI-4 in ASR-inducing activity. The mouse ASR assay was performed using 0.1% DMSO, 10 μM A23187, or 200 μM DDI-4. Data are shown as histograms indicating the mean ± SEM (*n* = 3). **, *P* < 0.01; ***, *P* < 0.005.

DDI-4 is a benzylamine analog (1-(4-*n*-pentylphenyl)ethan-1-amine; Fig 1A) with previously unknown biological activity. As shown in Fig 1B and 1C, under optimized assay conditions, MOF induction by DDI-4 reached a plateau at 100 µM and after 15 min of incubation, respectively. Then, we compared MOF-inducing activities of DDI-4 and its analogs (DDI-4A, 1-(4-ethylphenyl)ethan-1-amine; DDI-4B, (*S*)-(-)-1-phenylethylamine; and DDI-4C, (*R*)-(-)-1-phenylethylamine). Only DDI-4 triggered MOF in spermatids, suggesting that a specific alkyl chain length linked to the phenyl group is required for the MOF-inducing activity (Fig 1D).

When spermatids from males raised at high temperature (25°C) were examined, the ΔMOF-positive ratio induced by DDI-4—calculated by subtracting the MOF-positive ratio of the control sample from that of the DDI-4–treated sample—was markedly reduced to 17.9%, compared with 96.7% in spermatids raised at 20°C (Figs S2 and 1E). In contrast, ΔMOF-positive ratios by Pron or ProK were unaffected by the tested temperatures (Figs S2 and 1E). These results indicate that DDI-4, but neither Pron nor ProK, triggers temperature-sensitive MOF.

### DDI-4 activates the ASR and induces IZUMO1 relocation in mouse spermatozoa

We next tested whether DDI-4 activates the ASR in mouse spermatozoa (Fig 1F–1I). Acrosome (AS)-reacted spermatozoa were distinguished from AS-intact spermatozoa by staining acrosomal contents with Coomassie Brilliant Blue G-250 (Fig 1F). As in Fig 1B and 1C, the effects of DDI-4 concentration and incubation period on the ASR were examined; ASR-positive ratios approached a plateau at 200 µM DDI-4 (Fig 1G) or after 30 min of incubation (Fig 1H). Under these optimized conditions, ASR-positive ratios induced by DDI-4 were comparable to those induced by the calcium ionophore A23187, used as a positive control (Fig 1I).

**Fig 2:**
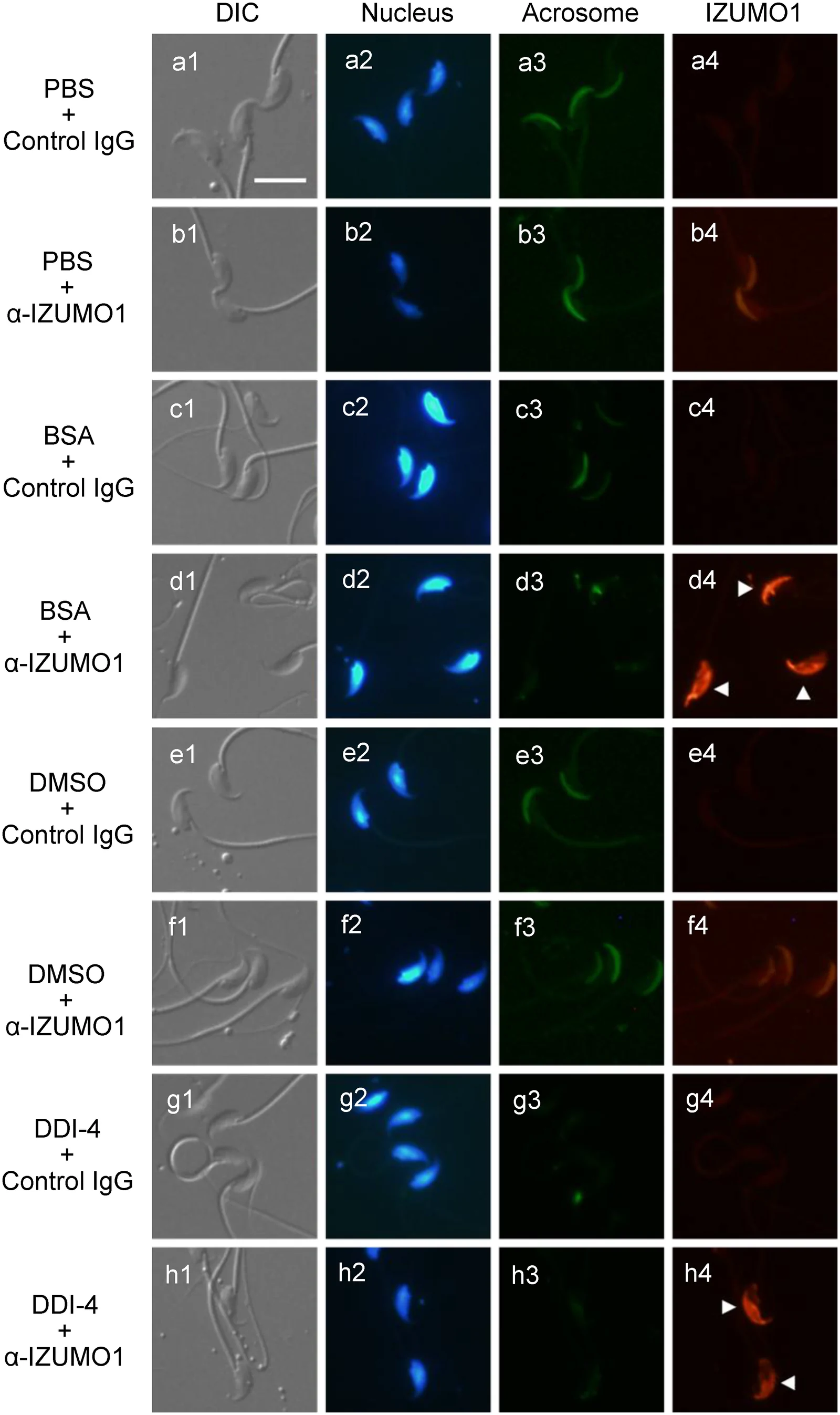
DDI-4 triggers IZUMO1 translocation during the ASR in mouse spermatozoa. Mouse spermatozoa were released from the cauda epididymis, incubated with 200 µM DDI-4, and stained with 5 µg/mL rabbit anti-mouse IZUMO1 antibody (α-IZUMO1). The sperm samples were also counterstained with 5 µg/mL 4′,6-diamidino-2-phenylindole (DAPI) and 5 µg/mL fluorescein isothiocyanate–labeled peanut agglutinin (FITC-PNA) to label the nucleus and the AS, respectively. We also used 3% bovine serum albumin (BSA) as a positive ASR inducer. PBS, phosphate-buffered saline; control IgG, normal rabbit IgG. Scale bar = 10 µm.

We also examined IZUMO1 relocation during DDI-4–induced ASR by immunofluorescence (IF) using a rabbit anti-mouse IZUMO1 polyclonal antibody (α-IZUMO1) [14] (Fig 2, column 4). The same samples were counterstained with 4′,6-diamidino-2-phenylindole (DAPI) (Fig 2, column 2) and fluorescein isothiocyanate–conjugated peanut agglutinin (FITC-PNA) (Fig 2, column 3) to visualize the nucleus and the AS, respectively.

When AS-intact spermatozoa were mock-treated with phosphate-buffered saline (PBS) as a solvent control, FITC-PNA stained the AS region of the sperm head (Fig 2, panels a3 and b3), whereas α-IZUMO1 immunoreactivity was not detected (Fig 2, panels a4 and b4), indicating that neither the ASR nor IZUMO1 relocation occurred. In the presence of bovine serum albumin (BSA), a conventional ASR inducer, the FITC-derived signals were diminished or absent in many spermatozoa (Fig 2, panels c3 and d3), indicating ASR induction. Concomitantly, α-IZUMO1 immunoreactive signals were detected in a region of the sperm head corresponding to the equatorial segment (Fig 2, panels c4 and d4), indicating IZUMO1 relocation during the ASR.

IF patterns observed with dimethyl sulfoxide (DMSO) (Fig 2, panels e1–f4) and DDI-4 (Fig 2, panels g1–h4) were similar to those obtained with PBS (Fig 2, panels a1–b4) and BSA (Fig 2, panels c1–d4), respectively. DDI-4–treated spermatozoa underwent the ASR (Fig 2, panels g3 and h3) and exhibited IZUMO1 relocation to the equatorial segment (Fig 2, panels g4 and h4). Thus, DDI-4 activates the ASR, demonstrating that DDI-4 targets are present in mouse spermatozoa as in nematode spermatids.

### DDI-4 activates an exocytotic sperm remodeling pathway distinguishable from canonical Pron- and ProK-mediated pathways

**Fig 3:**
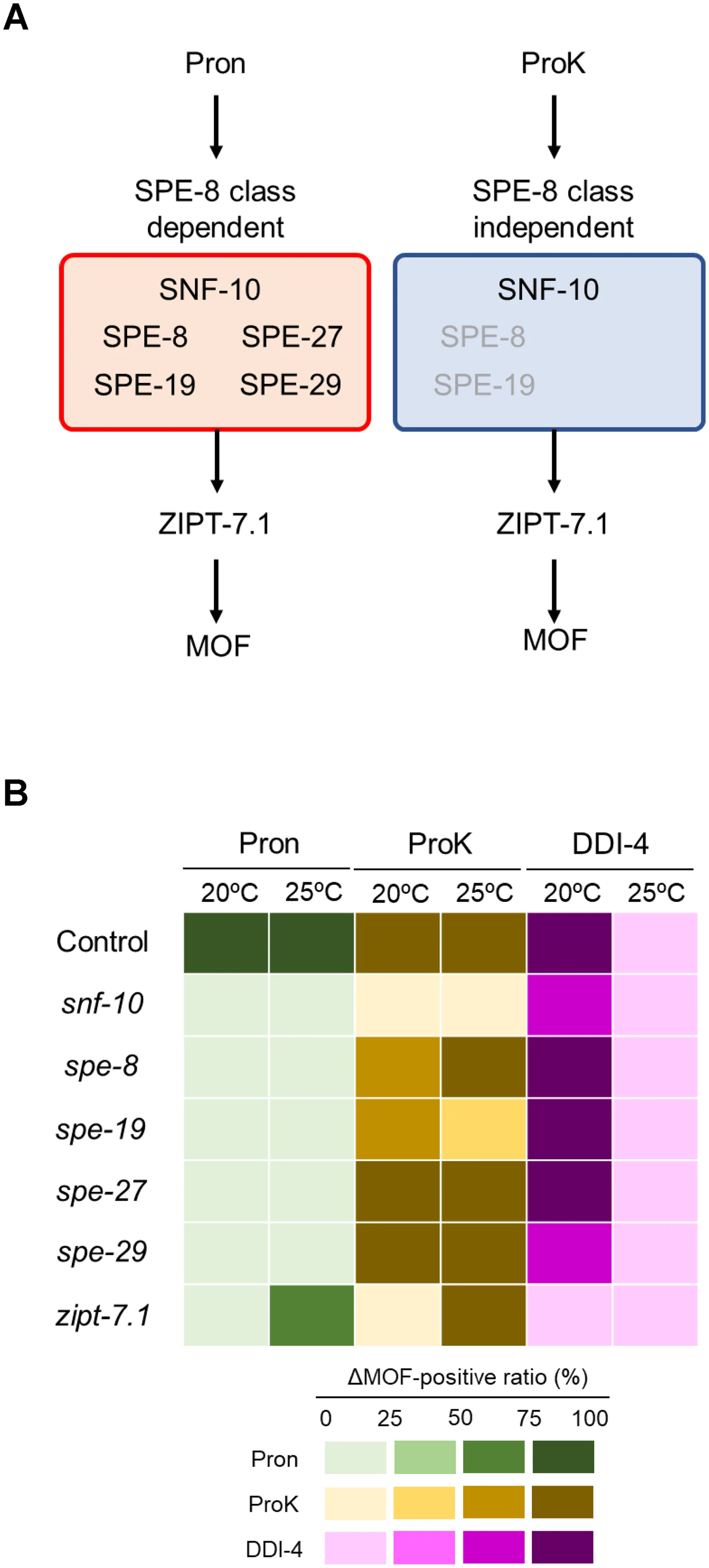
The DDI-4 pathway is distinct from the canonical exocytotic sperm remodeling pathways. **(A)** Canonical pathways activated by Pron or ProK. **(B)** Comparison of Pron-, ProK-, and DDI-4–induced MOF in spermatids from males lacking genes involved in the Pron and/or ProK pathways, raised at 20°C or 25°C. ΔMOF-positive ratios for each tested activator were calculated as described in Fig 1 and are shown as color gradients. The values of each ΔMOF-positive ratio are shown in S1 Table.

To elucidate the molecular basis of DDI-4–induced exocytotic sperm remodeling, we focused on the *C. elegans* system. Given that several genes involved in Pron- and/or ProK-mediated activation have been identified, we tested whether DDI-4 could activate spermatids carrying mutations in these genes. As shown in Fig 3A, previous studies have indicated that *snf-10* [10,15] and *zipt-7.1* [10,16] function in both the Pron and ProK pathways, whereas *spe-8* class genes (*spe-8* [9,10,17], *spe-19* [10,18], *spe-27* [10,19], and *spe-29* [10,20]) act primarily in the Pron pathway. Upon treatment with Pron or ProK, these mutant spermatids fail to undergo normal pseudopod extension and to exhibit the wild-type pattern of FM1-43 staining characteristic of MOF [10]. Our results (Fig 3B and S1 Table) were largely consistent with these profiles across the tested temperatures, except for the *zipt-7.1(ok971); him-5(e1490)* males: spermatids raised at 25°C exhibited higher ΔMOF-positive ratios than those raised at 20°C.

DDI-4 induced MOF in *spe-8(hc40)*, *spe-19(eb52)*, and *spe-27(it110)* spermatids raised at 20°C but failed to activate the *spe-27* mutant and significantly diminished activation of the *spe-8* and *spe-19* mutants when raised at 25°C (Fig 3B and S1 Table). *him-8(e1489); snf-10(hc194)* and *spe-29(it127)* spermatids exhibited moderate and minimal activation by DDI-4 at 20°C and 25°C, respectively (Fig 3B and S1 Table). In contrast, DDI-4–induced MOF was significantly reduced in *zipt-7.1* mutant spermatids at both temperatures (Fig 3B and S1 Table). These results suggest that *zipt-7.1* plays a significant role in DDI-4–induced MOF downstream of the DDI-4 target, whereas *snf-10* and *spe-29* likely contribute to lesser extents.

Furthermore, genetic comparisons of the activation profiles induced by Pron, ProK, and DDI-4 suggest that the *spe-8* class genes act downstream of the Pron target but upstream of the DDI-4 target. In the ProK-activated pathway, *snf-10* appears to function downstream of the ProK target but upstream of the DDI-4 target. Because Pron- and ProK-induced MOF was temperature-insensitive, unlike DDI-4–induced MOF (Figs S2 and 1E), the DDI-4–triggered pathway is likely at least partially distinct from those activated by Pron and ProK.

### *C*. e*legans* tRNA methyltransferase NSUN-2 is required for both meiosis and DDI-4–induced MOF

**Fig 4:**
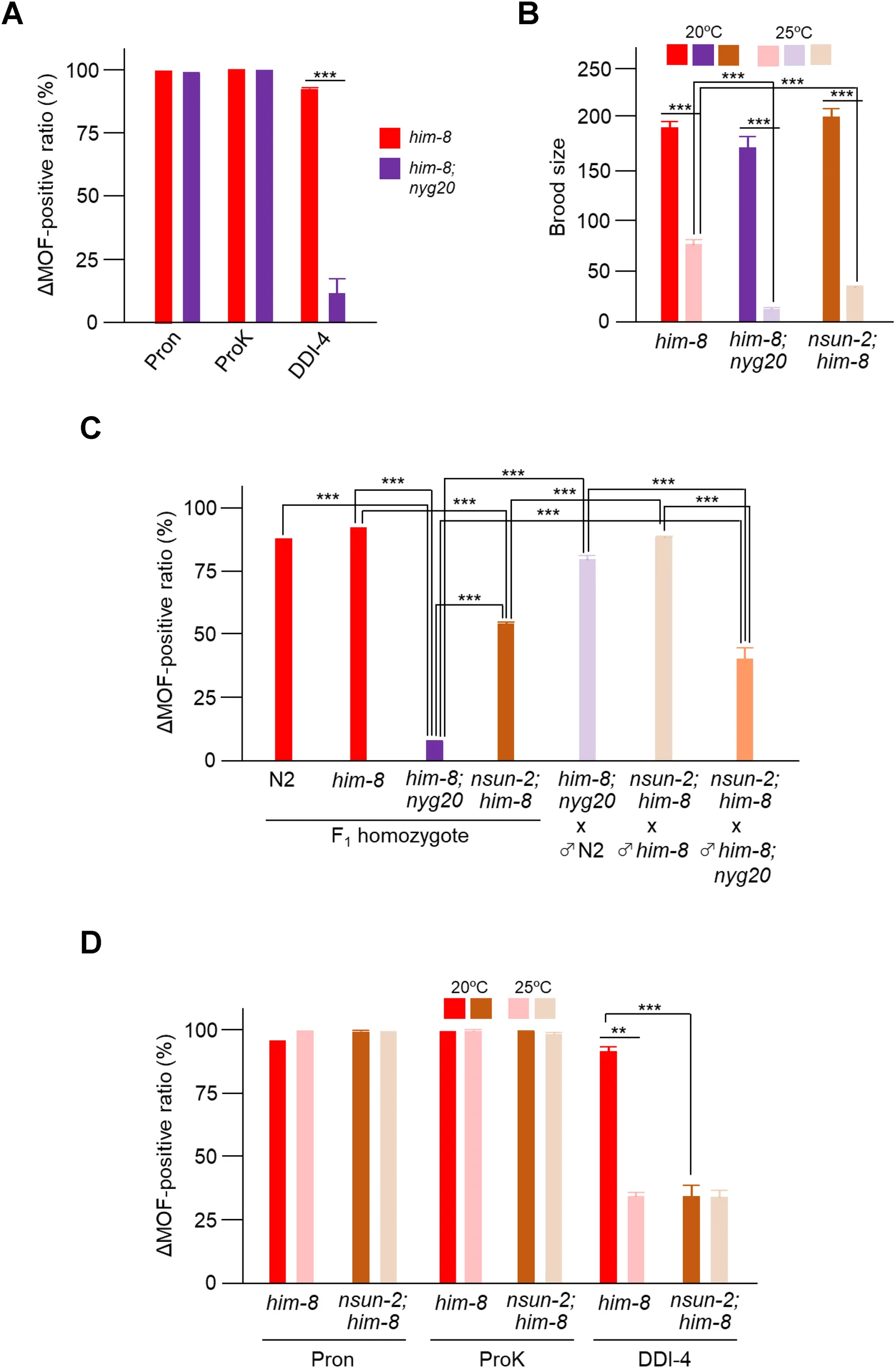
DDI-4–induced MOF are dependent on *nsun-2.* **(A)** MOF-inducing activity of DDI-4 in *nyg20* spermatids. ΔMOF-positive ratios were calculated as described in Fig 1 and are shown as histograms indicating the mean ± SEM (*n* = 3). Differences in ΔMOF-positive ratios between *him-8(tm611)* (control) and *him-8(tm611); nyg20* worms are indicated by asterisks. ***, *P* < 0.005. **(B)** Self-fertility of control and the *nyg20* and *nsun-2(nyg21)* hermaphrodites raised at 20°C or 25°C. Brood sizes of each strain are shown as histograms indicating the mean ± SEM (*n* = 3). ***, *P* < 0.005. **(C)** Complementation analysis between the *nyg20* and *nsun-2(nyg21)* worms. ΔMOF-positive ratios induced by DDI-4 were determined as described in Fig 1 and are shown as histograms indicating the mean ± SEM (*n* = 3). ***, *P* < 0.005. **(D)** Comparison of Pron-, ProK-, and DDI-4–induced MOF in spermatids from *him-8(tm611)* or *nsun-2(nyg21); him-8(tm611)* males raised at 20°C or 25°C. As described in Fig 1, ΔMOF-positive ratios were calculated, and statistical differences between net ratios are indicated by asterisks. **, *P* < 0.01; ***, *P* < 0.005.

To identify components involved in the DDI-4 pathway, we isolated the *nyg20* mutant after introducing random mutations into the *him-8(tm611)* genome using ethyl methanesulfonate (EMS). As shown in Fig 4A, *him-8(tm611); nyg20* males raised at 20°C produced spermatids that were Pron or ProK activated, but not by DDI-4, suggesting that this strain carries a mutation(s) affecting DDI-4–triggered MOF.

Our single-nucleotide polymorphism (SNP) mapping (S3 Fig and S2–S3 Tables) localized the *nyg20* mutation to a region near −6 cM on chromosome I and/or a region near −8 cM on chromosome X. During the SNP-mapping procedure, the *nyg20* mutant was crossed with the Hawaiian strain, and progeny homozygous for both *him-8(tm611)* on chromosome IV and the *nyg20* mutation were selected. Therefore, SNPs on chromosome IV were expected to be homozygous for the N2 allele in many of the analyzed clones. As part of our effort to identify the gene affected in *nyg20*, we focused on *nsun-2*, one of the genes located within this candidate interval, because it had previously been implicated in temperature-sensitive reproductive phenotypes. In *C. elegans*, NSUN-2 produced approximately 88% of total 5-methylcytosines (m^5^Cs) as a tRNA methyltransferase [21], and demethylation of these m^5^Cs by simultaneous disruption of all the *nsun* genes resulted in self-sterility at 27°C but not at 20°C [21]. These findings suggest that *C. elegans nsun-2* contributes to temperature-sensitive reproduction. Thus, we hypothesized that a subset of spermatid proteins translationally regulated by NSUN-2 functions during temperature-sensitive, DDI-4–induced MOF.

Using CRISPR/Cas9, we generated a predicted loss-of-function strain of *nsun-2* (*nsun-2(nyg21); him-8(tm611)*), carrying a premature stop codon in exon 2. As shown in Fig 4B, brood sizes of *him-8(tm611)*, *him-8(tm611); nyg20*, and *nsun-2(nyg21); him-8(tm611)* hermaphrodites raised at 20°C were comparable (~190, ~ 170, and ~200 F_1_ self-progeny per hermaphrodite, respectively). However, the *nyg20* and *nsun-2* mutants raised at 25°C exhibited markedly reduced brood sizes (~13 and ~34 F_1_ self-progeny) compared with control worms (~77 F_1_ self-progeny). This indicates that the *nyg20* and *nsun-2* mutants become severely sub-fertile at 25°C, but not at 20°C.

To determine whether *nsun-2* is affected in *nyg20*, complementation analysis was performed using the *nyg20* and *nsun-2* mutants raised at 20°C (Fig 4C). Male spermatids from self- or outcross progeny were subjected to the nematode MOF assay using DDI-4 as a SAF. Male F_1_ progeny from N2 (wild type), *him-8(tm611)* (control), *him-8(tm611); nyg20*, and *nsun-2(nyg21); him-8(tm611)* hermaphrodites produced spermatids exhibiting ΔMOF-positive ratios of 85.4, 92.1, 7.7, and 54.1%, respectively. Male outcross progeny heterozygous for the *nyg20* or *nsun-2* mutations displayed ΔMOF-positive ratios comparable to those of N2 and *him-8(tm611)* spermatids, respectively, indicating that both the *nyg20* and *nsun-2* mutations are recessive. Moreover, ΔMOF-positive ratios in male spermatids of outcross progeny from *nyg20*/*nsun-2* worms were comparable to those of the *nsun-2* mutant. Collectively, these results suggest that NSUN-2 function during DDI-4–induced MOF may be compromised in the *nyg20* mutant, although no mutations were identified in the *nsun-2* coding, intronic, and flanking regions by next-generation sequencing (NGS) or Sanger sequencing analyses (S4 Table).

In *Nsun2*-null mice [22], tRNA cleavage was increased, leading to reduced protein translation rates and activation of stress pathways. Because spermatogenesis is arrested at the spermatocyte stage in the *Nsun2* mutant mice, we also examined meiosis in the gonads of *nsun-2(nyg21); him-8(tm611)* male worms (S4 Fig). In *C. elegans* males, meiosis proceeds from the distal to proximal ends of the gonad (S4A Fig; *him-8(tm611)* (control) at 20°C). The distal mitotic region harbors germline stem cells, whereas the adjacent transition zone marks the onset of meiosis, where chromosome condensation produces crescent-shaped nuclei characteristic of the leptotene/zygotene stages. In control males raised at 20°C, crescent-shaped nuclei were detected in the transition zone by DAPI staining (S4A Fig). In contrast, in the *nsun-2* mutant, cells with crescent-shaped nuclei were mislocalized to the mitotic zone. Similar abnormalities were observed in the mitotic zone of control males raised at 25°C (S4A Fig). These findings suggest that male gonad meiosis is partially temperature-sensitive and *nsun-2*–dependent.

We also quantified the number of spermatids released from male gonads (S4B Fig). In single control males raised at 20°C, ~ 2,400 spermatids were obtained, whereas spermatid numbers were reduced by 42.2% and 32.9% in control males raised at 25°C and in *nsun-2* mutant males raised at 20°C, respectively. In the *nsun-2* mutant, rearing at 25°C further compromised spermatid production, with counts decreasing from ~1,600 cells at 20°C to ~1,000 cells. These findings suggest that meiosis is partially impaired by either elevated temperature or loss of NSUN-2 function. Intriguingly, the meiotic defects observed in the *nsun-2* mutant were prominent in males (S4 Fig), whereas self-fertility was largely unaffected in hermaphrodites (Fig 4B). Although the basis for this apparent sexual dimorphism remains unclear, one possible interpretation is that spermatogenesis occurs only during the fourth larval (L4) stage in hermaphrodites [2,3], whereas we examined the male germlines derived from adult males undergoing continuous spermatogenesis. Thus, the requirement for NSUN-2 may vary with the developmental stage or physiological context of the germline.

When spermatids from control and *nsun-2* mutant males were compared (Fig 4D), Pron or ProK treatment did not alter ΔMOF-positive ratios, regardless of genotype or rearing temperature. In contrast, the ratios of DDI-4–induced MOF-positive cells were significantly reduced by either the *nsun-2* mutation or elevated rearing temperature. These results indicate that *nsun-2* is required for temperature-sensitive MOF activation.

### DDI-4 is predicted to target sphingosine kinases (SPHKs)

**Fig 5:**
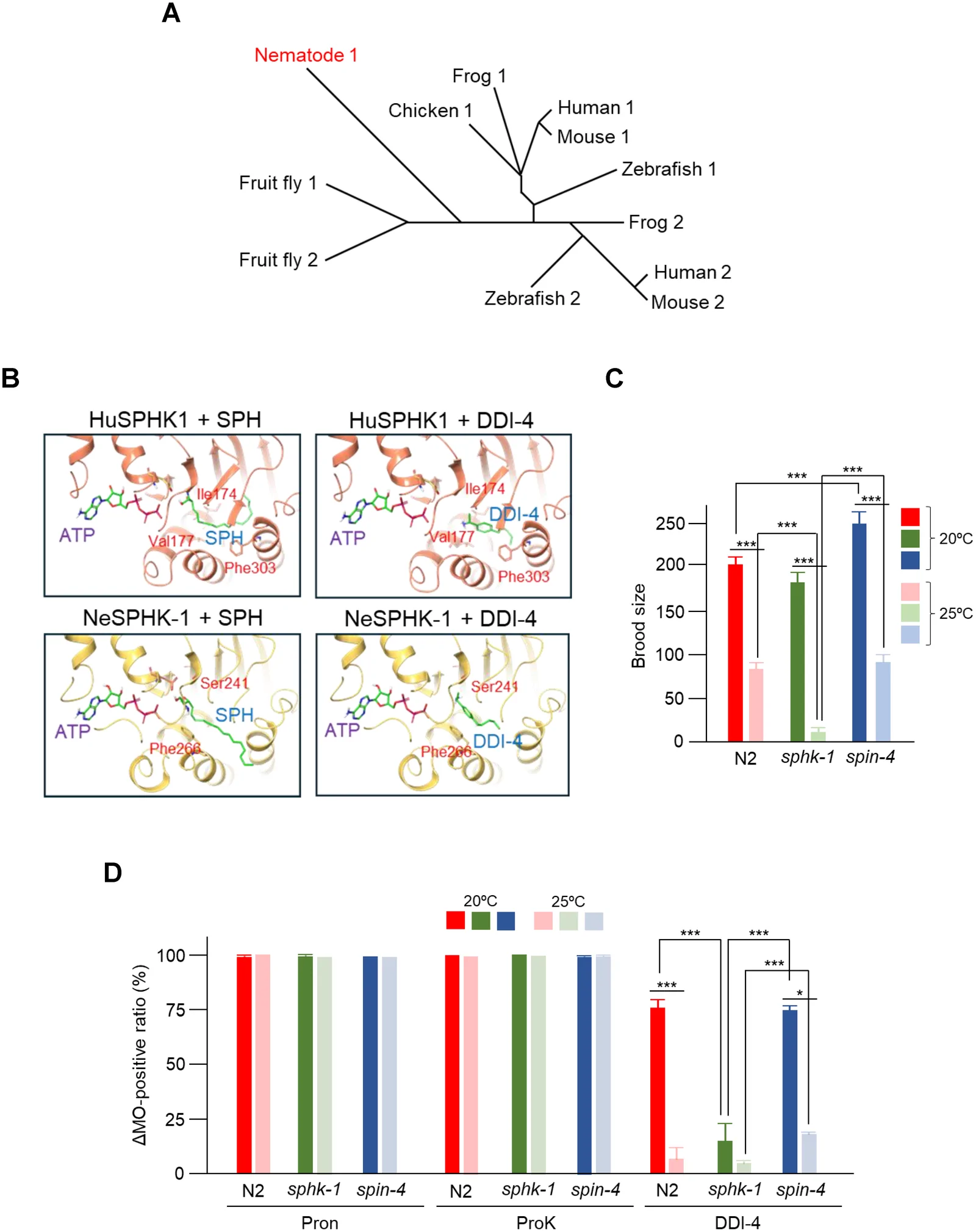
*sphk-1*, but not *spin-4*, is involved in DDI-4–induced MOF. **(A)** Phylogenetic analysis of sphingosine kinase (SPHK) homologs. A molecular phylogenetic tree was constructed using the neighbor-joining method implemented in MEGA11 (https://www.megasoftware.net/). The analysis was based on amino acid sequences of SPHKs retrieved from UniProt (https://www.uniprot.org/) and identified by their accession IDs: human SPHK1 (human 1; Q9NYA1); human SPHK2 (human 2; Q9NRA0); mouse SPHK1 (mouse 2; Q8CI15); mouse SPHK2 (mouse 2; Q9JIA7), chicken SPHK1 (chicken 1; A0A1D5P1F1), frog SPHK1 (frog 1; F7CBM1); frog SPHK2 (frog 2; A0A803JMT9); fruit fly SPHK1 (fruit fly 1; Q9VYY8); fruit fly SPHK2 (fruit fly 2; Q9VZW0); nematode SPHK-1 (nematode 1; Q18425). **(B)** Docking models of human SPHK1 (HuSPHK1) and nematode SPHK-1 (NeSPHK-1) with ATP and SPH or DDI-4. Note that Phe173 is obscured in the docking models of HuSPHK1, although this residue was predicted to participate in interactions with both SPH and DDI-4 (S6 Table). Phe173 is not visible in panel (B) because it is located behind the displayed structure. **(C)** Self-fertility of wild-type (N2), *sphk-1(ok1097)*, and *spin-4(knu1099)* hermaphrodites raised at 20°C or 25°C. Brood sizes of each strain are shown as histograms indicating the mean ± SEM (*n* = 3). ***, *P* < 0.005. **(D)** Comparison of Pron-, ProK-, and DDI-4–induced MOF in spermatids from N2, *sphk-1(ok1097)*, or *spin-4(knu1099)* males raised at 20°C or 25°C. As described in Fig 1, ΔMOF-positive ratios were calculated, and statistical differences in ΔMOF-positive ratios are indicated by asterisks. *, *P* < 0.05; ***, *P* < 0.005.

To understand how DDI-4 triggers MOF, it is essential to identify DDI-4 targets. *In silico* analysis using the Similarity Ensemble Approach (SEA; https://sea.bkslab.org/) [23] predicted that DDI-4 binds SPHKs (S5 Table). In vertebrates, SPHKs exist as two subtypes—SPHK1 and SPHK2 [24–27]. In contrast, *C. elegans* possesses a single SPHK homolog, SPHK-1. We conducted a phylogenetic analysis to infer which vertebrate subtype the *C. elegans* SPHK-1 is most closely related to. Fig 5A shows a phylogenetic tree of SPHK1 and SPHK2 proteins from various species, revealing two major vertebrate clades corresponding to SPHK1 and SPHK2. In contrast, invertebrate SPHKs (nematode SPHK-1 and fruit fly SPHK1/SPHK2) branched outside these clades, forming a distinct basal group that likely represents an ancestral SPHK lineage predating vertebrate gene duplication. Thus, this phylogenetic tree does not clearly classify nematode SPHK-1 as either vertebrate SPHK1 or SPHK2.

Next, based on X-ray crystallographic data of human SPHK1 in complex with ATP and sphingosine (SPH) [28,29], we constructed docking models of ATP and DDI-4 with human SPHK1 or nematode SPHK-1 (Fig 5B). These models revealed that the binding mode of DDI-4 to nematode SPHK-1 closely resembles that to human SPHK1. We predicted amino acid residues in human SPHK1 and nematode SPHK-1 that may mediate conserved interactions with both SPH and DDI-4 by the Protein-Ligand Interaction Profiler (PLIP) [30]. As shown in S6 Table, Phe173, Ile174, Val177, and Phe303 in human SPHK1 and Phe266 and Ser241 in nematode SPHK-1 appear to be involved in interactions with both SPH and DDI-4. Additionally, these models also predict that the alkyl chain length of DDI-4 is insufficient to properly fit the SPHK binding pocket, which may explain the relatively high DDI-4 concentration required to trigger MOF (Fig 1B).

We further examined whether the *C. elegans sphk-1* gene functions in the DDI-4–induced exocytotic sperm remodeling pathway. *spin-4(knu1099)* worms were also tested, as *spin-4* is a germline-specific gene encoding a transmembrane transporter for sphingosine-1-phosphate (S1P), which has been reported to act in spermiogenesis occurring in hermaphrodites [31].

When we compared the brood sizes of N2 (wild-type) and *sphk-1(ok1097)* hermaphrodites raised at 20°C or 25°C, those of the *sphk-1* mutant were severely reduced at 25°C but not at 20°C (Fig 5C). In contrast, *spin-4(knu1099)* hermaphrodites produced brood sizes comparable to those of wild type at 25°C, although their brood sizes at 20°C were higher than those of wild type (Fig 5C). We next compared Pron-, ProK-, and DDI-4–induced MOF in spermatids from wild-type, *sphk-1*, and *spin-4* mutant males raised at 20°C or 25°C. As observed in *nsun-2(nyg21)* males (Fig 4D), ΔMOF-positive ratios by Pron or ProK were unaffected by genotype or rearing temperature (Fig 5D). In contrast, when DDI-4 was used as a SAF, spermatids from *sphk-1* mutant males, but not from *spin-4* mutant males, raised at 20°C showed dramatically reduced MOF (Fig 5D). At 25°C, neither wild-type nor mutant spermatids responded to DDI-4 (Fig 5D). These results suggest that *sphk-1*, but not *spin-4*, is required for DDI-4–triggered exocytotic sperm remodeling.

**Fig 6:**
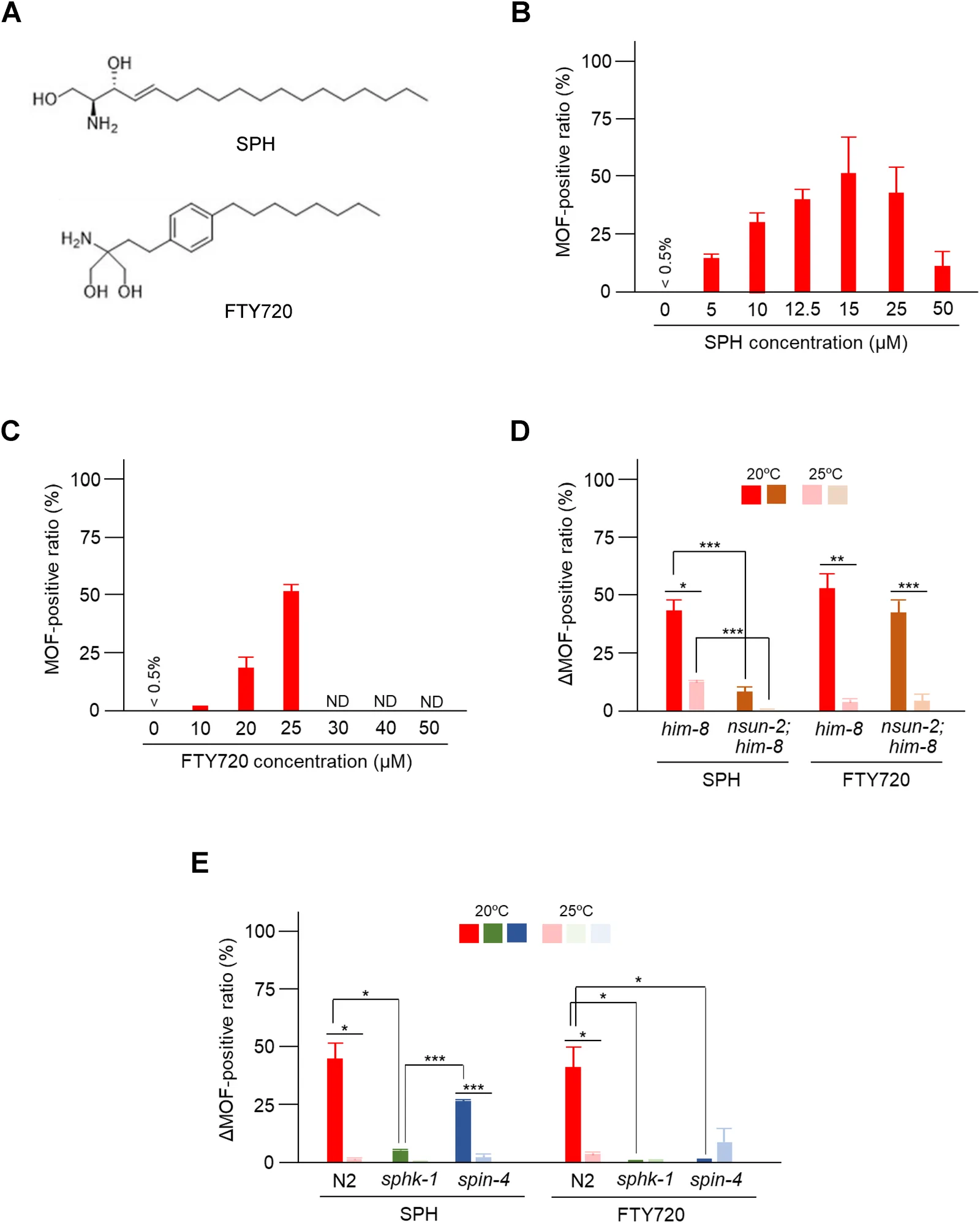
DDI-4 and sphingosine may induce MOF *via* the same pathway. **(A)** Chemical structures of sphingosine (SPH) and its analog, FTY720. **(B)** and **(C)** Spermatids from control (*him-8(tm611)*) males raised at 20°C were incubated at room temperature for 15 min with various concentrations of SPH **(B)** or FTY720 **(C)**. MOF-positive ratios were then measured. In panel **(C)**, FTY720 concentrations above 30 µM could not be evaluated because of precipitation. MOF-positive ratios in panels **(B)** and **(C)** were calculated from at least three independent experiments. ND, not determined. **(D)** *nsun-2*–dependence of MOF activation by SPH or FTY720. Spermatids from *him-8(tm611)* or *nsun-2(nyg21); him-8(tm611)* males raised at 20°C or 25°C were activated by 15 µM SPH or 25 µM FTY720. ΔMOF-positive ratios for each tested SAF were then calculated as described in Fig 1 (*n* ≥ 3). *, *P* < 0.05; **, *P* < 0.01; ***, *P* < 0.005. **(E)** Spermatids from N2, *sphk-1(ok1097)*, or *spin-4(knu1099)* males raised at 20°C or 25 °C were activated by 15 µM SPH or 25 µM FTY720. ΔMOF-positive ratios for each tested SAF were then calculated as described in Fig 1 (*n* ≥ 3). *, *P* < 0.05; ***, *P* < 0.005.

To confirm the involvement of SPH in the DDI-4–activated pathway, SPH and its analog FTY720 (Fig 6A) were tested for their ability to activate the DDI-4 pathway. MOF induced by SPH (Fig 6B) or FTY720 (Fig 6C) was concentration-dependent. In spermatids from *nsun-2(nyg21); him-8(tm611)* males raised at 20°C or 25°C, ΔMOF-positive ratios induced by SPH were significantly reduced compared to those in *him-8(tm611)* spermatids (Fig 6D). In contrast, FTY720-induced activation was unaffected by the *nsun-2* mutation or rearing temperature (Fig 6D). These results suggest that both SPH and FTY720 possess SAF activity, but only SPH-induced activation was *nsun-2*–dependent and temperature-sensitive.

Although SPH stimulated spermatids from *sphk-1(ok1097)* or *spin-4(knu1099)* males, the *sphk-1* mutation markedly reduced the proportion of MOF-positive sperm at both 20°C and 25°C, whereas *spin-4* mutant spermatids exhibited similar activation profiles to those of N2 spermatids regardless of rearing temperature (Fig 6E). The activation profile of FTY720 differed from that of SPH; FTY720 failed to activate spermatids regardless of genotype or rearing temperature (Fig 6E). Thus, SPH-triggered activation was *sphk-1*–dependent but *spin-4*–independent, similar to DDI-4. In contrast, FTY720 activated spermatids in a manner dependent on both *sphk-1* and *spin-4*, suggesting that FTY720 is mechanistically distinct from DDI-4 and SPH.

In a widely proposed model of SPH-mediated signaling, S1P receptors are present on the PM, and intracellularly generated S1P is exported *via* S1P transporters into the extracellular space, where it binds these receptors [32–37]. However, it remains unclear whether S1P receptors are encoded in the *C. elegans* genome. We therefore examined the subcellular localization of SPIN-4 in spermatids of the COP2416 strain, a CRISPR/Cas9-generated knock-in strain that is otherwise wild type and carries an mCherry tag inserted immediately upstream of the endogenous *spin-4* stop codon. As shown in S5 Fig, SPIN-4::mCherry signals co-localized with Alexa Fluor 488–labeled wheat germ agglutinin (Alexa 488-WGA), which labels MOs [38], suggesting that SPIN-4 localizes to the MO membrane. Thus, FTY720 appears to activate MOF through an organelle-intrinsic signaling pathway mediated by MO-localized SPIN-4.

### *nsun-2* and *sphk-1* may function genetically upstream of the DDI-4 pathway

We next examined whether spermatids from *him-8(e1489); snf-10(hc194)*, *zipt-7.1(ok971); him-5(e1490)*, *nsun-2(nyg21); him-8(tm611)*, and *sphk-1(ok1097)* males raised at 20°C or 25°C could be activated by alternative *in vitro* SAFs, including the weak base triethanolamine (TEA) [38], the cation ionophore monensin (MON) [9,39], and the chloride channel blocker 4,4′-diisothiocyanostilbene-2,2′-disulfonic acid (DIDS) [40] (S6A Fig).

S6B Fig and S7 Table show the activation profiles of spermatids from each mutant strain treated with TEA, MON, or DIDS. *snf-10* functions in both Pron- and ProK-induced activation pathways (Fig 3B and S1 Table). *snf-10* mutant spermatids showed a dramatically reduced response to TEA at both 20°C and 25°C. In contrast, MON or DIDS induced MOF in *snf-10* mutant spermatids raised at 20°C or 25°C, although MON-induced ΔMOF-positive ratios were reduced at 25°C. The *zipt-7.1* mutant, which likely function in both the DDI-4 pathway and canonical pathways (Fig 3B), similarly showed involvement in TEA responsiveness at both temperatures. Because DDI-4–induced MOF depends on *nsun-2* (Fig 4D) and *sphk-1* (Fig 5D), these genes may function upstream of the TEA target in the DDI-4–induced pathway. MON-induced activation was markedly attenuated in spermatids from *zipt-7.1* mutant males raised at 20°C and 25°C, whereas other tested mutations did not substantially affect MON-induced MOF at either rearing temperature. Only the *zipt-7.1* mutation also impaired DIDS-induced MOF in spermatids from males raised at 25°C. Thus, the MON and DIDS targets may not be essential for the DDI-4 pathway. Moreover, *zipt-7.1* presumably plays a crucial role in both canonical (Pron- and ProK-induced) and non-canonical (DDI-4–induced) exocytotic sperm remodeling pathways.

## Discussion

### NSUN-2 may be a key regulator of temperature-sensitive MOF

We isolated the *nyg20* mutant, in which spermatids fail to respond to DDI-4–induced activation (Fig 4A). Complementation analysis suggested that impaired *nsun-2* function contributes to the *nyg20* phenotype (Fig 4C), although no mutations were detected in the *nsun-2* coding, intronic, or flanking regions in the *nyg20* genome (S4 Table). Consistent with this observation, SNP mapping localized the causative mutation(s) in *nyg20* to chromosome I and/or chromosome X (S3 Fig and S2–S3 Tables). Because *nsun-2* is located on chromosome I, one possible interpretation is that *nyg20* affects an X-linked gene functionally related to *nsun-2*. Such a gene would be expected to escape X-chromosome silencing during spermatogenesis, unlike most X-linked genes [41]. Intriguingly, DDI-4–induced MOF was more severely impaired in *nyg20* than in the *nsun-2* single mutant (Fig 4C), raising the additional possibility that, besides affecting NSUN-2 function, *nyg20* may also compromise one or more genes that participate in DDI-4–induced MOF. Identification of the causative mutation(s) in *nyg20* will therefore be essential for distinguishing among these possibilities.

The male germline–acting genes translationally regulated by NSUN-2 remain unknown. However, our findings suggest that these genes are involved in meiosis (S4 Fig) and exocytotic sperm remodeling (Fig 4D). In *Nsun2*-null mice, male meiosis is arrested at the pachytene stage [22]. Owing to the severe meiotic defects in *Nsun2*-deficient male mice, which lead to markedly reduced spermatozoon production, it is challenging to determine whether *Nsun2*-null spermatozoa can undergo the ASR in response to DDI-4. Therefore, identifying *C. elegans* genes translationally regulated by NSUN-2 may provide insight into conserved mechanisms underlying meiosis and exocytotic sperm remodeling in nematodes and mice.

### MOF may be activated *via* at least three distinct pathways in *C. elegans* spermatids

Because DDI-4 is water-insoluble, it is likely that one or more intracellular proteins in *C. elegans* spermatids bind DDI-4 and transmit signals that induce temperature-sensitive, *nsun-2*–dependent MOF. This is consistent with the *in silico* analysis predicting intracellular SPHK-1 as a DDI-4 target (S5 Table) and with the genetic analysis exhibiting the involvement of *sphk-1* in DDI-4–induced MOF (Fig 5D).

In contrast, Pron and ProK are water-soluble SAFs, suggesting that these proteases generate extracellular signals that are transmitted to intracellular molecules to trigger MOF. Pron- or ProK-induced MOF was affected by mutations in genes encoding intracellular proteins, including *spe-8*, *spe-27*, and *zipt-7.1* (Fig 3B). However, Pron- or ProK-triggered exocytotic sperm remodeling was neither temperature-sensitive nor *nsun-2*–dependent (Fig 4D), genetically suggesting that DDI-4 activated a pathway at least partially distinct from those activated by Pron or ProK. Because *zipt-7.1* mutant spermatids failed to be activated by Pron, ProK, or DDI-4 (Fig 3B), these pathways may converge genetically at or near *zipt-7.1*. At any rate, the identification of physiological SAFs is essential for elucidating how each MOF-activation pathway is regulated.

The Pron and ProK pathways are considered to be activated by hermaphrodite- and male-produced SAFs *in vivo*, respectively, thereby suggesting that these two are canonical and essentially required for reproduction in *C. elegans*. In contrast, the DDI-4 pathway appears dispensable for nematode reproduction under standard rearing conditions. The biological significance of the DDI-4 pathway remains to be elucidated. However, one compelling hypothesis posits that this pathway emerged incidentally from the canonical Pron or ProK pathways and subsequently evolved as a non-canonical mechanism contributing to the ASR in mammalian spermatozoa. This proposition is noteworthy for identifying conserved molecular frameworks underlying exocytotic sperm remodeling in nematodes and mammals. Comparative analyses of the Pron/ProK and DDI-4 pathways will be important for delineating their respective contributions to ASR induction.

### SPH-mediated signaling is involved in temperature-sensitive MOF

Activation profiles of additional *in vitro* SAFs provided insight into the DDI-4–induced pathway. For instance, the weak base TEA, which increases intracellular pH [38], induced temperature-sensitive MOF by acting at a site genetically downstream of the DDI-4 target (S6 Fig and S7 Table). Because increased intracellular pH elevates intracellular Ca² ⁺ concentration ([Ca²⁺]_i_) in mammalian spermatozoa [42–46], SPH-mediated signaling may regulate [Ca²⁺]_i_ to promote MOF.

In a canonical model, SPH is phosphorylated by SPHKs to generate S1P, which is exported *via* S1P transporters and binds to G protein–coupled S1P receptors, initiating signaling cascades that regulate a variety of biological processes [32–37]. In *C. elegans*, loss of the S1P transporter gene *spin-4* appears to disrupt spermiogenesis in hermaphrodites, resulting in defective pseudopod extension [31]. However, in hermaphrodites, the SPIN-4 localization was not examined in spermatids, whereas SPIN-4 was detected on the self-sperm PM [31]. Because the MO membrane fuses with the spermatid PM during exocytotic sperm remodeling (S1 Fig), our observation that SPIN-4 localized to the MO membrane in male spermatids is not conflicted with the previous findings in hermaphrodites. Although *spin-4* involvement in MOF remains unclear, our findings indicate that *spin-4* is not essential for MOF induced by Pron, ProK, DDI-4, or SPH (Figs 5D and 6E). In contrast, FTY720 activated MOF in a SPIN-4–dependent manner (Fig 6E). SPHK2 preferentially phosphorylates FTY720, and the phosphorylated SPH analog binds S1P receptors [47,48]. However, it remains unclear whether FTY720 is a better substrate for *C. elegans* SPHK-1 in spermatids than SPH. Notably, SPH may activate MOF independently of phosphorylation, consistent with the observation that DDI-4, which lacks phosphorylation sites (Fig 1A), triggered MOF in an SPHK-1–dependent but SPIN-4–independent manner (Fig 5D), similar to SPH (Fig 6E). One possible interpretation is that *C. elegans* SPHK-1 functions as a scaffold for downstream signaling rather than solely as a catalytic kinase, when DDI-4 or SPH serves as a SAF.

Mitochondrial localization and the ability to phosphorylate FTY720 are characteristic of mammalian SPHK2 [27]. *C. elegans* SPHK-1 localizes to mitochondria [35] and likely phosphorylates FTY720 (Fig 6E), suggesting functional similarity to SPHK2. In general, it remains unclear whether FTY720 is a preferred substrate of SPHK2 relative to SPH. However, if nematode SPHK-1 preferentially phosphorylates FTY720 rather than SPH, this could explain why DDI-4– and SPH-induced MOF occurred independently of SPIN-4 (Figs 5D and 6E), whereas FTY720-induced MOF required SPIN-4 (Fig 6E).

Previous studies [49,50] have suggested that mammalian SPHK2 functions as a catalytic enzyme that phosphorylates SPH and FTY720 and as a scaffold that organizes signaling complexes on intracellular membranes. For instance, mitochondrial SPHK2 generates S1P, which binds Prohibitin 2 to regulate assembly of respiratory complex IV and mitochondrial respiration [49]. SPHK2 also activates autophagy by forming a complex with B-cell lymphoma 2 (BCL2), thereby dissociating the Beclin-1/BCL2 complex [50]. These findings suggest that SPHK2 acts as a molecular platform that spatially coordinates lipid signaling with protein–protein interactions, linking sphingolipid metabolism to mitochondrial function. Consistent with this notion, mitochondria-derived vesicles, termed mitophers, are expelled from developing *C. elegans* spermatids [51]. This process, known as mitopherogenesis, is a mitochondria-specific form of ectocytosis that regulates the mitochondrial number within cells. By modulating mitochondrial abundance, mitopherogenesis fine-tunes sperm motility and fertilization competence.

**Fig 7:**
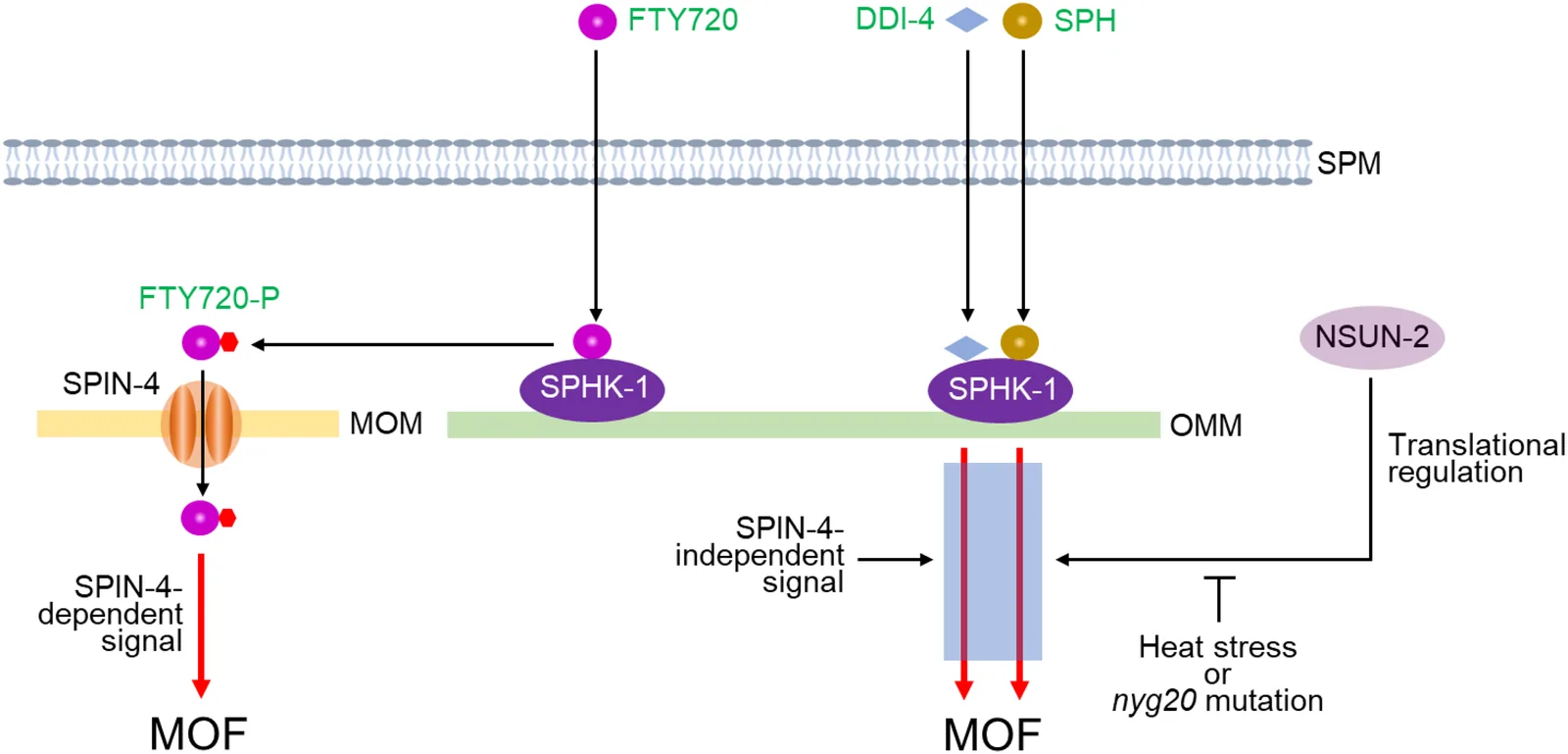
Model for the DDI-4–triggered exocytotic sperm remodeling pathway. SPM, spermatid plasma membrane; MOM, membranous organelle membrane; OMM, outer membrane of mitochondria; FTY720-P, phosphorylated FTY720. Created in BioRender. Nishimura, H. (2026) https://BioRender.com/7ramet3.

Fig 7 shows a proposed model for MOF activation by DDI-4, SPH, or FTY720, based on our findings. If SPHK-1 in *C. elegans* spermatids functions as a scaffold on the outer membrane of mitochondria, this could reconcile its apparent signaling activity with the absence of S1P production during MOF activation by DDI-4 or possibly SPH. Thus, the scaffold hypothesis provides a unifying explanation for the dual enzymatic and non-enzymatic functions of SPHKs across species. Once S1P is produced within spermatids, it may also induce MOF *via* SPIN-4 localized on the MO membrane.

In mammals, SPH-mediated signaling is presumably involved in the ASR [52]. S1P may promote the ASR by mobilizing [Ca²⁺]_i_ [53], implicating SPHKs in this process. Moreover, in mouse and capacitated human spermatozoa, SPHK1 localizes to the acrosomal region [53,54]. Although mice lacking either *Sphk1* [55] or *Sphk2* [56] remain fertile, double knockout of both genes results in embryonic lethality [57]. Hence, the genetic involvement of SPHK1 and SPHK2 in the ASR remains unclear. Our genetic analyses place NSUN-2 upstream of DDI-4–dependent MOF, but the molecular link between NSUN-2–mediated RNA methylation and sphingolipid signaling remains unknown. Clarifying this connection will be an important subject for future studies.

## Conclusions

Nematodes diverged from mammals approximately 708 million years ago [58], and reproductive genes evolve more rapidly than somatic genes [59–61]. Even among mammals, some testis-specific genes that are essential for fertilization in mice exist only as pseudogenes in humans [62,63]. Thus, identifying conserved mechanisms underlying reproductive processes across such evolutionarily distant species is challenging. Nevertheless, in the context of exocytotic sperm remodeling, the nematode *fer-1* [64] and the mouse *Fer1l5* [65] play analogous roles in MOF and ASR, respectively. Our integrative approach, combining pharmacological and genetic methods, is effective for identifying shared mechanisms of exocytotic sperm remodeling in both species. Our findings further suggest that mouse NSUN2 may regulate translation of male germline–acting genes in a temperature-sensitive manner, contributing to both the ASR and meiosis. Comparative analyses of this type provide insight into conserved principles governing reproduction across evolutionarily distant animals.

## Materials and methods

Key resources used in this study, including reagents and animals, are listed in S8 Table.

### Ethics statement

All nematode and mouse experiments were performed according to protocols approved by the Institutional Animal Care and Use Committee (IACUC) at Setsunan University (Approval Number K25-35).

### Animals

We maintained and cultured worms at 20°C unless otherwise specified, following previously described protocols [66]. For heat-stressed conditions, worms at the L4 stage were cultured at 25°C for 48 h. Most strains used in this study were obtained from the *Caenorhabditis* Genetics Center (CGC; University of Minnesota) or the National BioResource Project (NBRP; Tokyo Women’s Medical University). The *nyg20* mutant was isolated in this study by screening for genes involved in DDI-4–triggered MOF after introducing random mutations into the *him-8(tm611)* background by EMS mutagenesis (for details, see “EMS mutagenesis”). The *him-8* mutant hermaphrodites are otherwise normal but produce ~37% males among their self-progeny [67]. The *nyg20* mutant was outcrossed five times with the *him-8* mutant worms before use, to minimize unrelated background mutations. The *nsun-2(nyg21)* strain was generated by co-injecting synthetic single guide RNA, recombinant Cas9 nuclease, and a synthetic single-stranded DNA repair template targeting the *nsun-2* locus into the germline of *him-8(tm611)* hermaphrodites (for details, see ”CRISPR/Cas9”). Worms carrying the expected mutation in exon 2 of *nsun-2*, which introduces a premature stop codon and an EcoRI-cleavage site, were identified by single-worm PCR (SWP)–restriction fragment length polymorphism (RFLP) analysis (for details, see “SWP and SWP-RFLP analyses for genotyping”). A complete list of *C. elegans* strains is shown in S8 Table. On the other hand, to evaluate ASR-positive ratios in mouse spermatozoa, we used ~10-week-old Slc:ICR males (Japan Slc).

### Microscopy

Most worm handling procedures, such as picking and dissection, were performed under SZ61 or SZX10 stereomicroscopes (Evident). Digital differential interference contrast (DIC) and fluorescent images were captured using either a BX53 microscope equipped with a DP72 CCD camera and cellSens software (Evident) or an LSM 710 confocal microscope with ZEN 2010 software (Carl Zeiss). Co-localization of the SPIN-4::mCherry and Alexa 488–WGA signals was also observed and analyzed using the confocal microscope. The number of spermatids released from male gonads was quantified, following DAPI staining, using a BZ-X810 microscope equipped with BZ-X Analyzer software and BZ-H4A/BZ-H4C modules (Keyence).

### Nematode MOF assay

This assay was carried out essentially as previously described [10,13,68], and all steps were performed at room temperature (~22°C). Males at the L4 stage were cultured for 48 h at either 20°C or 25°C and then dissected to release round spermatids in 10 μL of 1x Sperm Medium (1x SM; 50 mM Hepes-NaOH, pH 7.4, containing 45 mM NaCl, 5 mM KCl, 1 mM MgSO_4_, 5 mM CaCl_2_, and 10 mM glucose). After 5-min incubation, 10 μL of SAF Solution (1x SM or 1x SM containing 400 μg/mL Pron, 400 μg/mL ProK, or 100 mM TEA; 1x SM containing 1% DMSO, 40–400 μM DDI-4, 200 μM DDI-4 analogs, or 1 mM DIDS; 1x SM containing 1% ethanol or 2 μM MON; 1x SM containing 1% dimethyl formamide, 10–100 μM SPH, or 20–100 μM FTY720) was added to the cells, and they were incubated for 5–15 min to induce spermiogenesis. To stain cellular membranes, 5 μL of FM1-43 Solution (1x SM containing 25 μg/mL FM1-43) was added to the 20-μL preparation. After a further 5-min incubation, digital DIC and fluorescent (FM1-43) images were acquired as described in “Microscopy”. The number of MOF-positive cells was counted from fluorescent images obtained from each experimental group.

### Mouse ASR assay

This assay was based on the previous report [69]. Spermatozoa were released from the cauda epididymis of ICR males into 200-μL drops of human tubal fluid omitting BSA medium (HTF(-) medium) and allowed to stand at 37°C for 30 min in 5% CO_2_/95% air. Then, 1.0 x 10^7^/mL epididymal spermatozoa were incubated with either of 0.1% DMSO (solvent control as a mock), 200 μM DDI-4, or 10 μM A23187 in 50 μL of HTF(-) medium at 37°C for 30 min in 5% CO_2_/95% air. To quench the ASR by fixing sperm, 100 μL of 1x PBS containing 4% paraformaldehyde (PFA) was added to the sperm suspension. After 20-min incubation at room temperature (~22°C), cells were washed three times by centrifugation at 2,200 x *g* for 5 min at room temperature in 200 μL of 100 mM ammonium acetate, pH 9.0, and then re-suspended in 100 μL of the same buffer. A 5-μL aliquot from the sperm suspension was put onto a glass slide and then air-dried at room temperature. To stain the AS in the air-dried spermatozoa with Coomassie Brilliant Blue G-250 (CBB), 50 μL of Staining Solution (0.22% CBB contained in 50% methanol and 10% acetic acid) was added to the air-dried cells. After 10 min of incubation at room temperature, cells were washed three times with ultrapure water in a chamber to remove excess CBB. The stained cells were mounted with 10 μL of 1x PBS and observed under a BX53 microscope, as described in “Microscopy”. To perform IF for IZUMO1, epididymal spermatozoa (1.0 x 10^7^/mL) were incubated with 1x PBS, 3% BSA, or 200 μM DDI-4 to induce the ASR. After fixation, washing, and air-drying as described above, the resulting sperm spots were washed three times with 50 μL of 1x PBS, and incubated with 50 μL of Blocking Solution (1x PBS containing 3% BSA and 0.05% Tween-20) for 30 min at room temperature. Then, the sperm spots were incubated with 50 μL of Primary Antibody Solution (Blocking Solution containing 5 µg/mL normal rabbit IgG or 5 µg/mL α-IZUMO1) for 60 min at room temperature, washed twice with Washing Solution (1x PBS containing 0.05% Tween-20), and incubated again with Secondary Antibody Solution (Blocking Solution containing 5 µg/mL Alexa Fluor 568–labeled goat anti-rabbit IgG antibody, 5 µg/mL FITC-PNA, and 5 µg/mL DAPI) for 30 min at room temperature. After washing three times with 50 μL of Washing Solution, one drop of ProLong antifade reagent was placed onto the sperm spots. Mouse spermatozoa were then observed under a BX53 microscope, as described in “Microscopy”.

### SWP and SWP-RFLP analyses for genotyping

For *him-8* or *nsun-2* genotyping, single worms were digested with 1 mg/mL ProK at 60°C for 60 min in 20 μL of 1x Phusion HF Buffer, followed by incubation at 100°C for 15 min to inactivate the enzyme. One microliter of each digest was used as a template in a 10-μL PCR reaction containing Phusion DNA polymerase and the primer pair STNHN326/STNHN327 for *him-8* or STNHN520/STNHN521 for *nsun-2*, according to the manufacturer’s instructions. The expected PCR fragment sizes for *him-8* were 2,105 bp for the wild-type allele and 1,692 bp for the *tm611* allele. To determine the *nsun-2* genotype in each worm, RFLP analysis of the PCR products was performed using EcoRI; the restriction digestion yielded a 314-bp fragment from the wild-type allele, and 241- and 82-bp fragments from the *nyg21* allele generated by CRISPR/Cas9 (see “CRISPR/Cas9”). For SNP mapping, DraI was used as a restriction enzyme for SWP-RFLP analysis (see “SNP mapping”).

### EMS mutagenesis

*C. elegans* hermaphrodites were mutagenized with EMS as described previously [10]. L4 *him-8(tm611)* hermaphrodites were incubated with 50 mM EMS at room temperature (~22°C) for ~4 h. Following mutagenesis, 10 P_0_ worms were placed on each 60-mm nematode growth medium (NGM) agar plate seeded with *E. coli* OP50 (20 plates; 200 P_0_ worms in total). After ~100 fertilized F_1_ eggs had been deposited on each plate, the P_0_ worms were removed and discarded. Twenty-five F_1_ hermaphrodites from each plate were individually transferred to fresh NGM plates (500 F_1_ worms in total). From each plate, ~ 20 F_2_ males were isolated and examined individually by the nematode MOF assay using DDI-4 to detect spermatids unresponsive to DDI-4. In total, approximately 10,000 F_2_ males were screened. When a positive F_2_ male was identified, additional males from the same pedigree were assayed until hermaphrodites producing exclusively DDI-4–resistant sons were established. From this screen, a single mutant line, *nyg20*, was isolated. Male spermatids from *nyg20* were unresponsive to DDI-4, whereas mutant hermaphrodites remained fertile at 20°C.

### SNP mapping

Many of the SNPs examined in this study were previously described [70], and SNP mapping was performed based on the methods reported in that study [70]. Briefly, *him-8(tm611); nyg20* hermaphrodites (P_0_) were outcrossed with males of the Hawaiian strain (CB4856). Approximately 20 L4 hermaphrodite progeny (F_1_) were individually transferred to 35-mm NGM plates (F_1_ plates). After confirming that each F_1_ plate contained approximately 100 fertilized eggs (F_2_), the F_1_ hermaphrodites were individually collected and subjected to SWP analysis for genotyping of *him-8* (see “SWP and SWP-RFLP analyses for genotyping”). From F_1_ plates containing F_1_ worms heterozygous for *him-8(tm611)*, a total of 200 L4-stage F_2_ hermaphrodites were individually transferred to NGM plates (F_2_ plates). From each F_2_ plate, approximately five F_3_ males were individually examined using the nematode MOF assay with DDI-4 as a SAF (see “Nematode MOF assay”). Finally, ten F_2_ plates in which all tested F_3_ males produced spermatids unresponsive to DDI-4 were selected as mapping plates. Males from each mapping plate were then subjected to SWP-RFLP analysis using DraI to genotype the SNPs listed in S2 Table. PCR primers used to amplify genomic regions containing these SNPs are listed in S8 Table.

### CRISPR/Cas9

A mixture containing 17.5 μM *nsun-2* crRNA, 17.5 μM tracrRNA, 17 μM Cas9 nuclease, and 1 μM STNHN519 was prepared according to the manufacturer’s instructions. To introduce the desired mutation into the *nsun-2* gene, the mixture was microinjected into the distal gonad arms of *him-8(tm611)* hermaphrodites at the young adult or adult stage. F_1_ self-progeny from the injected P_0_ worms were then subjected to SWP-RFLP analysis to identify animals carrying the mutation (see “SWP and SWP-RFLP analyses for genotyping”). Through subsequent self-fertilization, we established the *nsun-2(nyg21); him-8(tm611)* strain, which was used for further experiments.

### Complementation analysis

L4 hermaphrodites of *C. elegans* strains—wild-type (N2), *him-8(tm611)*, *him-8(tm611); nyg20,* and *nsun-2(nyg21); him-8(tm611)*—were cultured at 20°C for 48 h. F_1_ males at the L4 stage were individually picked from each NGM plate containing either of the strains, cultured at 20°C for 48 h, and subjected to the nematode MOF assay using DDI-4 as a SAF. In addition, *nyg20* and *nsun-2* mutant hermaphrodites at the L4 stage were outcrossed with adult N2 and *him-8(tm611)* males, respectively, at 20°C for 48 h. F_1_ male progeny obtained from these crosses were individually cultured at 20°C for 48 h and dissected to release spermatids for the nematode MOF assay using DDI-4. Male carcasses were subsequently collected for genotyping. F_1_ males derived from the outcrosses of N2/*nyg20* and *him-8(tm611)*/*nsun-2* were analyzed by SWP for the *tm611* allele and by SWP-RFLP for the *nyg21* allele, respectively.

### Evaluation of meiosis in the male gonad

Nuclei of male germ cells in the distal gonad were observed under an LSM 710 confocal microscope to assess meiotic progression. Briefly, to isolate gonads, males cultured for 48 h after the L4 stage at 20°C or 25°C were dissected in 5 μL of 1x M9 (22 mM KH_2_PO_4_, 42 mM Na_2_HPO_4_, 86 mM NaCl, and 1 mM MgSO_4_) containing 0.1% Tween-20 and 12 mM levamisole. After a 5-min incubation in 10 μL of 1x  M9 containing 1% PFA, the gonads were permeabilized by freeze-cracking [71] and incubated at room temperature for 1 min in 5 μL of 100% ethanol. Following three washes with 100 μL of 1x  PBS, the gonads were incubated at room temperature for 5 min in 5 μL of 1x  PBS containing 0.5% Triton X–100 and 1 mM EDTA. Subsequently, 5 μL of 1x  M9 containing 1 μg/mL DAPI was added, and the samples were incubated for an additional 5 min at room temperature. DAPI fluorescence was then observed under an LSM 710 confocal microscope. In addition, the number of spermatids was counted after release from the male gonad. L4 males were cultured for 48 h at 20°C or 25°C and then individually dissected in a 20-μL drop of 1x SM containing 5 μg/mL DAPI and 0.05% Tween-20 on a slide glass to release spermatids from the gonad. The number of cells was counted as described in “Microscopy.”

### Construction of docking models for SPHKs bound to ATP and either DDI-4 or SPH

The human SPHK1 (HuSPHK1)/ADP/SPH complex structure was modeled by superimposing the HuSPHK1/SPH complex (3VZB) and the HuSPHK1/ADP complex (3VZD). The ADP was manually modeled into the ATP-binding site to obtain the HuSPHK1/ATP/SPH complex structure. The initial nematode SPHK-1 (NeSPHK-1)/ATP/SPH complex structure was obtained by superimposing ATP and SPH of the HuSPHK1/ATP/SPH complex onto the NeSPHK-1 structure from the AlphaFold database [72]. The NeSPHK-1/ATP/SPH complex used as the docking template was obtained by minimizing the structure around the binding site of the initial complex. Docking of DDI-4 was performed using AutoDock Vina 1.2.5 [73]. The binding site center was defined as the center of mass of SPH, and a 30 Å cubic box was used to generate 10 binding poses. The final docking model was selected visually from the obtained binding poses. As shown in S6 Table, the amino acids involved in ligand interactions were identified using the Protein-Ligand Interaction Profiler (PLIP) [30]

### Evaluation of SPIN-4 localization in spermatids

In COP2416 worms, the mCherry gene was inserted in the N2 genetic background before the termination codon of the endogenous *spin-4* gene to express the SPIN-4::mCherry protein. L4 males of the N2 or the COP2416 strains were cultured for 48 h at 20°C and then dissected to release round spermatids in 10 μL of 1x SM. After 5-min incubation, spermatids were further incubated for 5 min in 10 μL of 1x SM containing 2% PFA to fix the cells. Following permeabilization by 5-min incubation in 10 μL of 1x SM containing 0.05% Triton X–100, the cells were stained by 5-min incubation in 10 μL of 1x SM containing 1 μg/mL DAPI and 5 μg/mL Alexa 488–WGA to visualize nuclei and MOs, respectively. Fluorescent images were acquired and analyzed as described in “Microscopy”.

### Quantification and statistical analysis

Statistical analyses were performed using Microsoft Excel. Percentage data were subjected to arcsine square-root transformation prior to analysis using Welch’s *t*-test. For all tests, *n* indicates the number of independent experiments (at least three), and at least 100 cells were analyzed per experiment. Statistical significance was defined as *P* < 0.05. All *P*-values are indicated in the figure legends by asterisks.

## Supporting information

S1 Text Supporting Materials and Methods. (DOCX)

S1 Fig Schemes of exocytotic sperm remodeling in nematodes and mice are comparable. **(A)** Membranous organelle fusion (MOF) in nematodes. MO, membranous organelle; PM, plasma membrane; SAF, spermatid-activating factor. **(B)** The acrosome reaction (ASR) in mice. OAM, outer acrosomal membrane; AS, acrosome; IAM, inner acrosomal membrane; EQS, equatorial segment. Panels **(A)** and **(B)** were prepared with reference to a previous report [74].
(TIF)

S2 Fig MOF induction by DDI-4, but not by conventional proteases, is temperature-sensitive. Spermatids were released from *him-8(tm611)* males raised at 20°C or 25°C, stimulated with 0.5% dimethyl sulfoxide (DMSO), 200 μg/mL Pronase (Pron), 200 μg/mL Proteinase K (ProK), or 100 μM DDI-4, and then stained with 1 μg/mL FM1-43, a lipophilic styryl dye. ΔMOF-positive ratios in Fig 1E were calculated based on these micrographs. DIC, differential interference contrast. Scale bar = 10 µm.
(TIF)

S3 Fig Single-nucleotide polymorphisms (SNPs) used for identifying the *nyg20* mutation. **(A)** Genetic positions of the SNPs examined in this study. Genetic map positions (cM) were obtained from WormBase (WS298). **(B)** Comparison of DraI restriction fragment patterns between the N2 and Hawaiian (HW) strains for genomic regions containing each SNP. More detailed information is provided in S2 Table. Note that some DNA fragments are not visible in the gel images because they migrated beyond the region presented in the images. Chr., chromosome.
(TIF)

S4 Fig Meiotic defects in male gonads caused by the *nsun-2* mutation or elevated temperature. **(A)** Gonads were dissected from *him-8(tm611)* or *nsun-2(nyg21); him-8(tm611)* males raised at 20°C or 25°C. After fixation with 1% paraformaldehyde (PFA), male gonads were stained with 1 µg/mL 4′,6-diamidino-2-phenylindole (DAPI), and fluorescent signals were observed using an LSM 710 confocal microscope. White arrowheads indicate crescent-shaped nuclei, some of which are cleared from the gonads as apoptotic cells. M, mitotic region; T, transition zone. Scale bar = 20 µm. **(B)** Left: Images of spermatids released from single males of *him-8(tm611)* or *nsun-2(nyg21); him-8(tm611)* worms raised at 20°C or 25°C. Scale bar = 100 µm. Right: The number of spermatids quantified from the micrographs appeared on the left. Data are shown as histograms indicating the mean ± standard error of the mean (SEM; *n* = 3). *, *P* < 0.05; **, *P* < 0.01.
(TIF)

S5 Fig SPIN-4 localizes to MOs in nematode spermatids. **(A)** Spermatids were released from males of the wild-type (N2) strain or the COP2416 strain, in which SPIN-4::mCherry is endogenously expressed, then fixed and permeabilized. The cells were simultaneously stained with 1 μg/mL DAPI and 5 μg/mL Alexa Fluor 488–labeled wheat germ agglutinin (Alexa 488–WGA) to visualize nuclei and MOs, respectively. mCherry signals were subsequently observed. Scale bar = 10 μm. **(B)** From the Alexa 488–WGA and mCherry images shown in panel **(A)**, fluorescence intensity profiles were extracted along the indicated lines (10 μm in length) to examine whether SPIN-4 localizes to MOs in spermatids. Green and pink lines represent Alexa 488–WGA and SPIN-4::mCherry signals, respectively.
(TIF)

S6 Fig Activation profiles of MOF by conventional compounds. **(A)** Chemical structures of triethanolamine (TEA), monensin (MON), and 4,4′-diisothiocyanostilbene-2,2′-disulfonic acid (DIDS). **(B)** Comparison of TEA, MON, and DIDS in MOF-inducing activity toward spermatids from males lacking *snf-10*, *zipt-7.1*, *nsun-2*, or *sphk-1*, raised at 20°C or 25°C. ΔMOF-positive ratios for each tested spermatid-activating factor were calculated by subtracting the baseline values obtained with 1x Sperm Medium, 0.5% ethanol, and 0.5% DMSO from those obtained with 50 mM TEA, 1 μM MON, and 500 μM DIDS, respectively, and are shown as color gradients. The values of each ΔMOF-positive ratio are shown in S7 Table.
(TIF)

S7 Fig ^1^H NMR (A) and ^13^C NMR (B) spectra of Compound A (1-(4-*n*-pentylphenyl)ethan-1-ylidenehydroxylamine). (TIF)

S8 Fig ^1^H NMR (A) and ^13^C NMR (B) spectra of DDI-4 (1-(4-*n*-pentylphenyl)ethan-1-amine). (TIF)

S9 Fig ^1^H NMR (A) and ^13^C NMR (B) spectra of Compound B (1-(4-ethylphenyl)ethan-1-ylidenehydroxylamine). (TIF)

S10 Fig ^1^H NMR (A) and ^13^C NMR (B) spectra of DDI-4A (1-(4-ethylphenyl)ethan-1-amine). (TIF)

S1 Table Relevance of the DDI-4–triggered exocytotic sperm remodeling pathway to the canonical, protease-induced pathways. (DOCX)

S2 Table SNPs used for identifying the *nyg20* mutation. (DOCX)

S3 Table Summary for SNP mapping of the *nyg20* mutation. (DOCX)

S4 Table Sequencing analysis of *nsun-2* in the *nyg20* mutant. (DOCX)

S5 Table *In silico* prediction of DDI-4 targets. (DOCX)

S6 Table Predicted conserved interactions between sphingosine kinases (SPHKs) and sphingosine (SPH) or DDI-4. (DOCX)

S7 Table Comparison of TEA-, MON-, and DIDS-induced MOF in spermatids from MOF-related mutants. (DOCX)

S8 Table Key resources used in this study. (DOCX)

S1 Data Numerical data underlying the graphs presented in the figures. (XLSX)
