# Supplementary material for: DDI-4 induces a temperature-sensitive exocytotic remodeling during C. elegans spermiogenesis via nsun-2–dependent sphingosine signaling
Source: PLoS Genet. 2026 Aug 27;22(8):e1012275. doi: 10.1371/journal.pgen.1012275 (PMC13521401; doi:10.1371/journal.pgen.1012275)
Supplement: Supplementary file 1 [file pgen.1012275.s001.docx]

**Supporting Materials and Methods**

**Synthesis of DDI-4 and DDI-4A**

Nuclear magnetic resonance (¹H NMR (400 MHz) and ¹³C NMR (100 MHz)) spectra were recorded on a JEOL ECZ 400S spectrometer. Chemical shifts for ¹H NMR are reported in δ (ppm) relative to tetramethylsilane as an internal standard, and coupling constants (*J*) are given in hertz (Hz). The following abbreviations are used for multiplicities: s = singlet, d = doublet, t = triplet, m = multiplet, and br = broad. Chemical shifts for ¹³C NMR are reported in δ (ppm) relative to the center line of the CDCl_3_ triplet at 77.16 ppm. High-resolution mass spectrometry (HRMS) was performed on a JEOL JMS-700T mass spectrometer equipped with an electron impact (EI) ionization source. Analytical thin-layer chromatography (TLC) was performed on precoated silica gel 60 F_254_ plates (Merck, 0.25 mm thick). Flash column chromatography was performed on SiliaFlash P60 silica gel, unless otherwise specified. All reagents were of commercial grade and used without further purification unless otherwise stated.

*Synthesis of Compound A (1-(4-n-pentylphenyl)ethan-1-ylidenehydroxylamine)*

To a solution of 1-(4-n-pentylphenyl)ethan-1-one (600 µL, 3.0 mmol) in 80% ethanol (15 mL) were added sodium acetate (490 mg, 6.0 mmol) and hydroxylamine hydrochloride (330 mg, 4.9 mmol). After the reaction mixture was stirred under reflux for 2 h, the solvent was evaporated under reduced pressure. The residue was dissolved in diethyl ether, and the organic layer was washed with water and dried over anhydrous sodium sulfate (Na_2_SO_4_). After filtration, the filtrate was concentrated under reduced pressure. The crude product was purified by column chromatography (hexane/ethyl acetate = 9:1) to afford Compound A (580 mg, 94%) as a white solid. Also see S7 Fig.

^1^H NMR (400 MHz, CDCl_3_) δ: 0.89 (3H, t, *J* = 7.0 Hz), 1.25–1.39 (4H, m), 1.57–1.69 (2H, m), 2.29 (3H, s), 2.61 (2H, t, *J* = 7.7 Hz), 7.19 (2H, d, *J* = 8.2 Hz), 7.54 (2H, d, *J* = 8.2 Hz), 8.72 (1H, br s).

^13^C NMR (100 MHz, CDCl_3_) δ: 12.3, 14.2, 22.7, 31.2, 31.6, 35.8, 126.1, 128.7, 134.0, 144.5, 156.2.

MS (EI) m/z = 205 (M⁺); HRMS (EI) calcd. for C_13_H_19_NO (M⁺): 205.1467, found: 205.1467.

*Synthesis of DDI-4 (1-(4-n-pentylphenyl)ethan-1-amine)*

A mixture of Compound A (400 mg, 2.0 mmol) and 10% Pd/C (210 mg, 0.20 mmol) in ethanol (9.8 mL) was hydrogenated under an atmosphere of hydrogen (1 atm) at room temperature. After being stirred for 3 h, the reaction mixture was filtered through a pad of Celite, and the filtrate was concentrated under reduced pressure. The crude product was purified by column chromatography (chloroform/methanol = 9:1) to afford DDI-4 (260 mg, 85%) as a yellow oil.

^1^H NMR (400 MHz, CDCl_3_) δ: 0.89 (3H, t, *J* = 6.8 Hz), 1.24–1.37 (4H, m), 1.39 (3H, d, *J* = 6.8 Hz), 1.54–1.66 (2H, m), 1.79 (2H, br s), 2.59 (2H, t, *J* = 7.5 Hz), 4.09 (1H, q, *J* = 6.8 Hz), 7.14 (2H, d, *J* = 7.9 Hz), 7.25 (2H, d, *J* = 7.9 Hz). Also see S8 Fig.

^13^C NMR (100 MHz, CDCl_3_) δ: 14.2, 22.7, 25.6, 31.4, 31.7, 35.7, 51.2, 125.7, 128.6, 141.7, 144.8.

MS (EI) m/z = 176, 191 (M⁺); HRMS (EI) calcd. for C_13_H_21_N (M⁺): 191.1674, found: 191.1671.

*Synthesis of Compound B (1-(4-ethylphenyl)ethan-1-ylidenehydroxylamine)*

To a solution of 1-(4-ethylphenyl)ethan-1-one (600 µL, 4.0 mmol) in 80% ethanol (20 mL) were added sodium acetate (660 mg, 8.0 mmol) and hydroxylamine hydrochloride (460 mg, 6.6 mmol). After the reaction mixture was stirred under reflux for 2 h, the solvent was evaporated under reduced pressure. The residue was dissolved in diethyl ether, and the organic layer was washed with water and dried over anhydrous sodium sulfate (Na_2_SO_4_). After filtration, the filtrate was concentrated under reduced pressure. The crude product was purified by column chromatography (hexane/ethyl acetate = 9:1) to afford Compound B (620 mg, 94%) as a white solid. Also see S9 Fig.

^1^H NMR (400 MHz, CDCl_3_) δ: 1.24 (3H, t, *J* = 7.6 Hz), 2.28 (3H, s), 2.67 (2H, q, *J* = 7.6 Hz), 7.21 (2H, d, *J* = 8.0 Hz), 7.55 (2H, d, *J* = 8.0 Hz), 7.76–7.89 (1H, br s).

MS (EI) m/z = 163 (M⁺); HRMS (EI) calcd. for C_10_H_13_NO (M⁺): 163.0997, found: 163.1003.

*Synthesis of DDI-4A (1-(4-ethylphenyl)ethan-1-amine)*

A mixture of Compound B (330 mg, 2.0 mmol) and 10% Pd/C (220 mg, 0.20 mmol) in ethanol (10 mL) was hydrogenated under an atmosphere of hydrogen (1 atm) at room temperature. After being stirred for 2 h, the reaction mixture was filtered through a pad of Celite, and the filtrate was concentrated under reduced pressure. The crude product was purified by column chromatography (chloroform/methanol = 4:1) to afford DDI-4A (260 mg, 85%) as a yellow oil. Also see S10 Fig.

^1^H NMR (400 MHz, CDCl_3_) δ: 1.23 (3H, t, *J* = 7.6 Hz), 1.38 (3H, d, *J* = 6.6 Hz), 1.72 (2H, br s), 2.64 (2H, q, *J* = 7.6 Hz), 4.09 (2H, q, *J* = 6.6 Hz), 7.17 (2H, d, *J* = 7.8 Hz), 7.26 (2H, d, *J* = 7.8 Hz).

^13^C NMR (100 MHz, CDCl_3_) δ: 15.7, 25.7, 28.6, 51.2, 125.8, 128.1, 142.9, 145.1.

MS (EI) m/z = 134, 149 (M⁺); HRMS (EI) calcd. for C_10_H_15_N (M⁺): 149.1204, found: 149.1207.
