# Supplementary material for: DDI-4 induces a temperature-sensitive exocytotic remodeling during C. elegans spermiogenesis via nsun-2–dependent sphingosine signaling
Source: PLoS Genet. 2026 Aug 27;22(8):e1012275. doi: 10.1371/journal.pgen.1012275 (PMC13521401; doi:10.1371/journal.pgen.1012275)
Supplement: Supplementary file 2 [file pgen.1012275.s012.docx]

**S1 Table. Relevance of the DDI-4–triggered exocytotic sperm remodeling pathway to the canonical, protease-induced pathways.**

|  | ΔMOF-positive ratio (%) | | | | | | | |
| --- | --- | --- | --- | --- | --- | --- | --- | --- |
|  | Pron | |  | ProK | |  | DDI-4 | |
|  | 20ºC | 25ºC |  | 20ºC | 25ºC |  | 20ºC | 25ºC |
| Control | 97.4 ± 1.3  (1,276) | 98.5 ± 0.6  (1,121) |  | 98.8 ± 0.3  (1,475) | 99.5 ± 0.0  (1,118) |  | 81.2 ± 2.5  (1,388) | 4.8 ± 3.8  (1,417) |
| *snf-10* | 0  (1,383) | 0  (1,152) |  | 0  (1,372) | 1.5 ± 1.1  (1,066) |  | 61.2 ± 8.6  (1,250) | 6.9 ± 2.3  (1,446) |
| *spe-8* | 4.8 ± 2.2  (1,071) | 7.2 ± 1.9  (1,232) |  | 51.8 ± 4.2  (1,138) | 90.1 ± 2.5  (1,107) |  | 95.6 ± 1.8  (1,393) | 4.7 ± 1.6  (1,185) |
| *spe-19* | 0  (1,190) | 0  (1,356) |  | 63.0 ± 11.6  (1,366) | 42.5 ± 0.9  (1,211) |  | 85.0 ± 2.8  (1,210) | 0.4 ± 0.1  (1,328) |
| *spe-27* | 1.1 ± 0.4  (1,399) | 1.3 ± 0.6  (1,267) |  | 85.6 ± 4.5  (1,454) | 92.9 ± 1.2  (937) |  | 87.5 ± 7.8  (1,553) | 0  (1,395) |
| *spe-29* | 4.2 ± 2.2  (1,364) | 13.8 ± 0.8  (1,004) |  | 99.5 ± 0.4  (1,777) | 87.8 ± 5.8  (1,380) |  | 58.3 ± 14.1  (616) | 2.8 ± 2.2  (1,210) |
| *zipt-7.1* | 8.3 ± 5.1  (929) | 72.9 ± 3.7  (1,343) |  | 12.7 ± 5.5  (514) | 87.7 ± 2.6  (1,120) |  | 15.2 ± 5.4  (580) | 7.3 ± 2.3  (1,041) |

ΔMOF-positive ratios are shown as mean ± SEM (*n* ≥ 3), and each number in parentheses indicates the total number of cells counted. For details, see the main text and the legend for Fig 3.
