# Supplementary material for: DDI-4 induces a temperature-sensitive exocytotic remodeling during C. elegans spermiogenesis via nsun-2–dependent sphingosine signaling
Source: PLoS Genet. 2026 Aug 27;22(8):e1012275. doi: 10.1371/journal.pgen.1012275 (PMC13521401; doi:10.1371/journal.pgen.1012275)
Supplement: Supplementary file 3 [file pgen.1012275.s013.docx]

**S2 Table. SNPs used for identifying the *nyg20* mutation.**

| Chr. | Genetic position  (cM) | Physical position  (Mb) | Gene containing SNP | Fragment size  after DraI digestion (bp) | |
| --- | --- | --- | --- | --- | --- |
|  |  |  |  | N2 | HW (CB4856) |
| I | −19 | 0.17 | *F56C11* | 354, 146 | 500 |
|  | −12 | 1.91 | *Y71G12A* | 503, 72 | 377, 126, 72 |
|  | −6 | 2.82 | *W03D8* | 395, 144 | 539 |
|  | −2 | 3.83 | *cewivar00073402* | 255, 165 | 420 |
|  | −1 | 4.59 | *D1007* | 325, 134, 41 | 459, 41 |
|  | +5 | 10.72 | *B0205* | 494 | 365, 129 |
|  | +13 | 12.05 | *F58D5* | 446 | 295, 11 |
|  | +14 | 12.73 | *T06G6* | 236, 99, 78 | 335, 78 |
|  | +26 | 14.68 | *Y105E8B* | 360, 114, 27 | 474, 27 |
| II | −6 | 3.03 | *F54D10* | 516 | 387, 129 |
|  | +11 | 12.61 | *Y38E10A* | 484 | 353, 131 |
| III | −12 | 2.60 | *Y71H2B* | 368, 105 | 473 |
|  | +12 | 11.66 | *Y41C4A* | 339, 156 | 495 |
| IV | −16 | 1.80 | *Y38C1BA* | 304, 187 | 491 |
|  | +8 | 13.05 | *F49E11* | 313, 77 | 390 |
| V | −17 | 1.77 | *F36H9* | 307, 87, 79 | 386, 87 |
|  | −13 | 2.73 | *C24B9* | 288, 167 | 455 |
|  | −5 | 4.55 | *Y61A9LA* | 454 | 397, 147 |
|  | +1 | 7.09 | *VC5* | 435, 70 | 300, 135, 70 |
|  | +6 | 13.95 | *R10D12* | 500 | 348, 152 |
|  | +10 | 16.32 | *F57G8* | 475 | 288, 187 |
|  | +13 | 17.61 | *Y6G8* | 282, 205 | 487 |
|  | +18 | 18.78 | *Y17D7B* | 324, 164 | 488 |
| X | −17 | 2.07 | *F49H12* | 540 | 321, 219 |
|  | −8 | 4.16 | *ZK470* | 422, 72, 40 | 326, 96, 72, 40 |
|  | −4 | 5.93 | *C46F4* | 169, 54, 51, 35, 22 | 223, 51, 35, 22 |
|  | +17 | 14.55 | *T24C2* | 409, 34 | 302, 107, 34 |

Genotypes of each SNPs were determined by single-worm PCR (SWP)–restriction fragment length polymorphism (RFLP) analysis using DraI. Also see S3 Fig and S3 Table.
