# Supplementary material for: DDI-4 induces a temperature-sensitive exocytotic remodeling during C. elegans spermiogenesis via nsun-2–dependent sphingosine signaling
Source: PLoS Genet. 2026 Aug 27;22(8):e1012275. doi: 10.1371/journal.pgen.1012275 (PMC13521401; doi:10.1371/journal.pgen.1012275)
Supplement: Supplementary file 4 [file pgen.1012275.s014.docx]

**S3 Table. Summary for SNP mapping of the *nyg20* mutation.**

| Chr. | Genetic  Position  (cM) | Clone No. | | | | | | | | | |
| --- | --- | --- | --- | --- | --- | --- | --- | --- | --- | --- | --- |
|  |  | 1 | 2 | 3 | 4 | 5 | 6 | 7 | 8 | 9 | 10 |
| I | −19 | N2/HW | N2/HW | HW | N2/HW | HW | N2 | N2 | N2 | N2 | N2 |
|  | **−12** | N2 | N2/HW | N2 | N2/HW | N2 | N2 | N2 | N2 | N2 | N2 |
|  | **−6** | N2 | N2 | N2 | N2 | N2 | N2 | N2 | N2 | N2 | n.d. |
|  | −2.01 | N2 | N2/HW | N2 | N2 | N2/HW | N2/HW | N2/HW | N2/HW | N2/HW | N2/HW |
|  | −1 | N2/HW | N2/HW | N2/HW | N2 | N2/HW | N2/HW | N2/HW | N2 | N2/HW | N2/HW |
|  | +5 | N2/HW | N2 | N2 | N2 | N2 | N2/HW | N2/HW | N2 | N2 | N2/HW |
|  | +13 | N2/HW | N2 | N2 | N2 | N2/HW | N2/HW | N2/HW | N2/HW | N2/HW | N2/HW |
| II | −6 | HW | N2/HW | N2/HW | N2/HW | HW | N2/HW | N2 | HW | HW | HW |
|  | +11 | HW | N2/HW | N2/HW | N2/HW | HW | HW | N2/HW | HW | N2/HW | HW |
| III | −12 | HW | N2/HW | HW | N2/HW | HW | HW | N2 | N2/HW | HW | N2/HW |
|  | +12 | HW | N2/HW | N2 | N2/HW | HW | HW | N2/HW | N2/HW | HW | HW |
| IV | **−16** | N2 | N2/HW | N2 | HW | N2 | N2 | N2 | N2 | N2 | N2 |
|  | **+8** | N2 | N2 | N2 | HW | N2/HW | N2 | N2 | N2 | N2 | N2 |
| V | −13 | HW | N2/HW | N2 | N2/HW | HW | N2/HW | N2/HW | HW | N2 | N2 |
|  | +13 | N2/HW | N2 | N2 | N2 | N2 | HW | HW | N2 | HW | N2/HW |
| X | −17 | N2 | N2/HW | N2 | N2/HW | N2 | n.d. | n.d. | n.d. | n.d. | n.d. |
|  | **−8** | N2 | N2 | N2 | N2 | N2 | N2 | N2 | N2 | HW | N2 |
|  | −4 | N2 | N2/HW | N2 | N2/HW | N2 | n.d. | n.d. | n.d. | n.d. | n.d. |
|  | +17 | N2 | N2/HW | N2 | N2/HW | N2 | HW | HW | HW | HW | N2 |

Genotypes homozygous for the N2 allele at each SNP are highlighted in red, and SNPs at which more than 80% of the tested clones were N2-homozygous are indicated in bold green text. Also see S3 Fig and S2 table. n.d., not determined.
