# Supplementary material for: DDI-4 induces a temperature-sensitive exocytotic remodeling during C. elegans spermiogenesis via nsun-2–dependent sphingosine signaling
Source: PLoS Genet. 2026 Aug 27;22(8):e1012275. doi: 10.1371/journal.pgen.1012275 (PMC13521401; doi:10.1371/journal.pgen.1012275)
Supplement: Supplementary file 5 [file pgen.1012275.s015.docx]

**S4 Table. Sequencing analysis of *nsun-2* in the *nyg20* mutant.**

| Region analyzed |  | Method |  | Result | |
| --- | --- | --- | --- | --- | --- |
| Coding region |  | NGS and Sanger |  | No mutation detected | |
| Intronic region |  | NGS |  | No mutation detected | |
| 5’ flanking region |  | NGS |  | No mutation detected | |
| 3’ flanking region |  | NGS |  | No mutation detected | |
| NGS, next-generation sequencing; Sanger, Sanger sequencing. | | | | |  |
