# Supplementary material for: DDI-4 induces a temperature-sensitive exocytotic remodeling during C. elegans spermiogenesis via nsun-2–dependent sphingosine signaling
Source: PLoS Genet. 2026 Aug 27;22(8):e1012275. doi: 10.1371/journal.pgen.1012275 (PMC13521401; doi:10.1371/journal.pgen.1012275)
Supplement: Supplementary file 6 [file pgen.1012275.s016.docx]

| Target protein | Species | *P*-Value |
| --- | --- | --- |
| Sphingosine kinase 1 | Human | 3.952e^-87^ |
| Sphingosine kinase 2 | Mouse | 1.152e^-68^ |
| Sphingosine kinase 2 | Human | 4.456e^-65^ |
| S1P receptor 4 | Human | 3.170e^-53^ |
| RAC-alpha serine/threonine-protein kinase | Mouse | 1.220e^-52^ |
| S1P receptor 5 | Human | 1.190e^-43^ |
| S1P receptor 2 | Human | 2.077e^-42^ |
| Uracil-DNA glycosylase | Human | 1.208e^-38^ |
| S1P receptor 3 | Human | 2.643e^-34^ |
| Phospholipase C gamma-1 | Bovine | 3.053e^-32^ |
| Trace amine-associated receptor 1 | Macmu | 1.852e^-29^ |
| Plasmepsin-2 | Plafa | 6.840e^-27^ |
| Toll-like receptor 8 | Human | 3.767e^-25^ |
| S1P receptor 1 | Human | 1.066e^-23^ |

**S5 Table. *In silico* prediction of DDI-4 targets.**

DDI-4–binding proteins were predicted using the Similarity Ensemble Approach (SEA; https://sea.bkslab.org/). S1P, sphingosine-1-phosphate; RAC, Ras-related C3 botulinum toxin substrate.
