# Supplementary material for: DDI-4 induces a temperature-sensitive exocytotic remodeling during C. elegans spermiogenesis via nsun-2–dependent sphingosine signaling
Source: PLoS Genet. 2026 Aug 27;22(8):e1012275. doi: 10.1371/journal.pgen.1012275 (PMC13521401; doi:10.1371/journal.pgen.1012275)
Supplement: Supplementary file 7 [file pgen.1012275.s017.docx]

**S6 Table. Predicted conserved interactions between sphingosine kinases (SPHKs) and sphingosine (SPH) or DDI-4.**

| Human SPHK1 | | | | | | |
| --- | --- | --- | --- | --- | --- | --- |
| SPH | | |  | DDI-4 | | |
| Residue | Distance (Å) | Interaction type |  | Residue | Distance (Å) | Interaction type |
| F173 | 3.96 | Hydrophobic |  | F173 | 3.69 | Hydrophobic |
| I174 | 3.81 | Hydrophobic |  | I174 | 3.67 | Hydrophobic |
| V177 | 3.73 | Hydrophobic |  | V177 | 3.69 | Hydrophobic |
| F192 | 3.45 | Hydrophobic |  | L200 | 3.82 | Hydrophobic |
| L261 | 3.93 | Hydrophobic |  | L259 | 3.68 | Hydrophobic |
| F288 | 3.84 | Hydrophobic |  | L268 | 3.48 | Hydrophobic |
| F303 | 3.98 | Hydrophobic |  | L299 | 3.71 | Hydrophobic |
| D81 | 2.77 | Hydrogen bond |  | F303 | 3.94 | Hydrophobic |
| S168 | 2.38 | Hydrogen bond |  | F303 | 3.73 | Hydrophobic |
| S168 | 2.60 | Hydrogen bond |  | D178 | 3.00 | Hydrophobic |
|  |  |  |  |  |  |  |
| Nematode SPHK-1 | | | | | | |
| SPH | | |  | DDI-4 | | |
| Residue | Distance (Å) | Interaction type |  | Residue | Distance (Å) | Interaction type |
| T26 | 3.30 | Hydrophobic |  | L240 | 3.87 | Hydrophobic |
| I250 | 3.76 | Hydrophobic |  | F266 | 3.83 | Hydrophobic |
| D251 | 3.45 | Hydrophobic |  | F266 | 3.61 | Hydrophobic |
| F266 | 3.77 | Hydrophobic |  | I375 | 3.71 | Hydrophobic |
| F266 | 3.97 | Hydrophobic |  | I375 | 3.91 | Hydrophobic |
| T267 | 3.87 | Hydrophobic |  | A376 | 3.94 | Hydrophobic |
| L413 | 3.79 | Hydrophobic |  | I415 | 3.76 | Hydrophobic |
| E416 | 3.96 | Hydrophobic |  | I415 | 3.72 | Hydrophobic |
| E416 | 3.81 | Hydrophobic |  | S241 | 3.25 | Hydrogen bond |
| D153 | 1.79 | Hydrogen bond |  | Y368 | 2.06 | Hydrogen bond |
| G186 | 3.34 | Hydrogen bond |  | F266 | 4.13 | π-Stacking |
| S241 | 3.38 | Hydrogen bond |  |  |  |  |
| D251 | 1.72 | Hydrogen bond |  |  |  |  |

In the docking models of SPHKs with SPH or DDI-4 (Fig 5B), amino acid residues that participate in the binding of both SPH and DDI-4 were predicted by the Protein-Ligand Interaction Profiler (PLIP) and are highlighted in red. Also see Fig 5B.
