# Supplementary material for: DDI-4 induces a temperature-sensitive exocytotic remodeling during C. elegans spermiogenesis via nsun-2–dependent sphingosine signaling
Source: PLoS Genet. 2026 Aug 27;22(8):e1012275. doi: 10.1371/journal.pgen.1012275 (PMC13521401; doi:10.1371/journal.pgen.1012275)
Supplement: Supplementary file 8 [file pgen.1012275.s018.docx]

|  | ΔMOF-positive ratio (%) | | | | | | | |
| --- | --- | --- | --- | --- | --- | --- | --- | --- |
|  | TEA | |  | MON | |  | DIDS | |
|  | 20ºC | 25ºC |  | 20ºC | 25ºC |  | 20ºC | 25ºC |
| Control | 57.3 ± 0.6  (1,954) | 17.5 ± 5.6  (699) |  | 61.6 ± 4.4  (1,070) | 53.2 ± 1.5  (1,539) |  | 80.4 ± 4.5  (1,289) | 96.0 ± 0.8  (760) |
| *snf-10* | 15.6 ± 4.8  (870) | 2.4 ± 0.5  (1,091) |  | 77.1 ± 1.1  (1,211) | 67.6 ± 3.3  (982) |  | 83.6 ± 2.9  (822) | 89.9 ± 2.1  (1,239) |
| *zipt-7.1* | 19.5 ± 2.4  (1,263) | 6.8 ± 0.9  (343) |  | 7.0 ± 2.4  (1,178) | 42.6 ± 5.5  (1,026) |  | 99.1 ± 0.3  (1,026) | 46.3 ± 10.5  (733) |
| *nsun-2* | 72.9 ± 1.0  (1,001) | 9.6 ± 0.5  (191) |  | 83.2 ± 3.7  (1,132) | 50.3 ± 2.2  (1,550) |  | 88.1 ± 2.7  (1,256) | 92.4 ± 0.5  (1,132) |
| *sphk-1* | 66.2 ± 5.2  (1,495) | 0.5 ± 0.3  (1,365) |  | 63.8 ± 1.7  (1,415) | 71.1 ± 2.1  (1,725) |  | 56.7 ± 8.6  (903) | 82.9 ± 5.6  (970) |

**S7 Table. Comparison of TEA-, MON-, and DIDS-induced MOF in spermatids from MOF-related mutants.**

ΔMOF-positive ratios are shown as mean ± SEM (*n* ≥ 3), and each number in parentheses indicates the total number of cells counted. For details, see the main text and the legend for S6 Fig.
