# Supplementary material for: DDI-4 induces a temperature-sensitive exocytotic remodeling during C. elegans spermiogenesis via nsun-2–dependent sphingosine signaling
Source: PLoS Genet. 2026 Aug 27;22(8):e1012275. doi: 10.1371/journal.pgen.1012275 (PMC13521401; doi:10.1371/journal.pgen.1012275)
Supplement: Supplementary file 9 [file pgen.1012275.s019.docx]

**S8 Table. Key resources used in this study.**

| Antibody | Source | Identifier |
| --- | --- | --- |
| Rabbit anti-mouse IZUMO1 antibody (α-IZUMO1) | Inoue et al., 2005 | N/A |
| Rabbit IgG, whole molecule | Pierce | Cat# 31235 |
| Alexa 568-labeled goat anti-rabbit IgG (H + L) antibody | Invitrogen | Cat# A-11004 |

| Bacterial strain | Source | Identifier |
| --- | --- | --- |
| *Eschericia coli*: Strain OP50 | *Caenorhabditis* Genetics Center (CGC) | RRID:WB-STRAIN: WBStrain00041969 |
| *Eschericia coli*: Strain DH5α (NEB5-alpha) | New England Biolabs (NEB) | Cat# C2987H |
| Chemical/peptide/protein | Source | Identifier |
| 1-(4-*n*-Pentylphenyl)ethan-1-amine (DDI-4) | This study | N/A |
| 1-(4-Ethylphenyl)ethan-1-amine (DDI-4A) | This study | N/A |
| (*S*)-(-)-1-Phenylethylamine (DDI-4B) | Tokyo Chemical Industry (TCI) | Cat# P0793 |
| (*R*)-(-)-1-Phenylethylamine (DDI-4C) | TCI | Cat# P0793 |
| Protease from *Streptomyces griseus*, Type XIV (Pron) | Sigma-Aldrich | Cat# P5147 |
| Proteinase K (ProK) | Nacalai Tesque | Cat# 29442-14 |
| Triethanolamine (TEA) | Sigma-Aldrich | Cat# 90279 |
| 4,4’-Diisothiocyanostilbene-2,2’-disulfonic acid (DIDS) | Sigma-Aldrich | Cat# D3514 |
| Monensin (MON) | TCI | Cat# M2573 |
| D-Sphingosine (SPH) | TCI | Cat# S0874 |
| Fingolimod (FTY720) | Cayman Chemical | Cat# 10006292 |
| FM1-43 dye | Invitrogen | Cat# T3163 |
| Paraformaldehyde (PFA), 16% aqueous solution | Electron Microscopy Sciences | Cat# 15710 |
| Coomassie Brilliant Blue G-250 (CBB) | Nacalai Tesque | Cat# 09409-42 |
| Albumin from bovine serum (BSA) | Sigma-Aldrich | Cat# A3733 |
| Calcium ionophore A23187 | Sigma-Aldrich | Cat# C7522 |
| 4',6-Diamidino-2-phenylindolen (DAPI) | Dojindo | Cat# D523 |
| Fluorescein isothiocyanate‒labeled peanut agglutinin  (FITC-PNA) | Sigma-Aldrich | Cat# L7381 |
| ProLong glass antifade mountant | Invitrogen | Cat# P36980 |
| Ethyl methanesulfonate (EMS) | Nacalai Tesque | Cat# 325-81522 |
| Phusion high‒fidelity DNA polymerase | NEB | Cat# M0530S |
| Alt-R S.p. Cas9 nuclease V3 | Integrated DNA Technologies (IDT) | Cat# 1081058 |
| Alexa Fluor 488‒labeled wheat germ agglutinin  (Alexa 488‒WGA) | Invitrogen | Cat# W11261 |
| EcoRI | NEB | Cat# R101S |
| DraI | NEB | Cat# R0129S |
| Medium | Source | Identifier |
| HTF medium omitting BSA (HTF(-) medium) | NK System | Cat# 9600-001 |
| Critical commercial assays | Source | Identifier |
| NucleoSpin Tissue | Takara | Cat# U0952S |
| Experimental animal | Source | Identifier |
| *M. musculus*: Slc:ICR: Wild type | Japan SLC | RRID:MGI:2160272 |
| *C. elegans*: Strain N2: Wild type | CGC | RRID:WB-STRAIN: WBStrain00000001 |
| *C. elegans*: Strain DR466: *him-5(e1490) V* | CGC | RRID:WB-STRAIN: WBStrain00006269 |
| *C. elegans*: Strain CA257: *him-8(tm611) IV* | National BioResource Project (NBRP) | RRID:WB-STRAIN: WBStrain00004044 |
| *C. elegans*: Strain HTN108: *nsun-2(nyg21) I;*  *him-8(tm611) IV* | This study | N/A |
| *C. elegans*: Strain HTN109: *him-8(tm611) IV; nyg20* | This study | N/A |
| *C. elegans*: Strain BA785: *spe-8(hc40) I* | CGC | RRID:WB-STRAIN: WBStrain00000389 |
| *C. elegans*: Strain AD213: *spe-19(eb52) V; asEx83* | CGC | RRID:WB-STRAIN: WBStrain00000052 |
| *C. elegans*: Strain BA900: *spe-27(it110) IV* | CGC | RRID:WB-STRAIN: WBStrain00000405 |
| *C. elegans*: Strain BA962: *spe-29(it127) IV* | CGC | RRID:WB-STRAIN: WBStrain00000410 |
| *C. elegans*: Strain HTN110: *him-8(e1489) IV;*  *snf-10(hc194) V* | This study | N/A |
| *C. elegans*: Strain HTN111: *zipt-7.1(ok971) IV;*  *him-5(e1490) V* | This study | N/A |
| *C. elegans*: Strain VC916: *sphk-1(ok1097) II* | CGC | RRID:WB-STRAIN: WBStrain00036171 |
| *C. elegans*: Strain COP2456: *spin-4(knu1099) III* | CGC | RRID:WB-STRAIN: WBStrain00062538 |
| *C. elegans*: Strain COP2416: *spin-4(knu1071[spin-4::*  *mCherry]) III* | CGC | RRID:WB-STRAIN: WBStrain00062537 |
| *C. elegans*: Strain CB4956 (Hawaiian): Wild type | CGC | RRID:WB-STRAIN: WBStrain00004602 |
| Oligonucleotide | Source | Identifier |
| *W03D8*_FW: 5’-GTTTTCACTTTTGCCGGTGT-3’ | Eurofins Genomics | STNHN203 |
| *W03D8*_RV: 5’-TGAAGGCGCATATACAGCAG-3’ | Eurofins Genomics | STNHN204 |
| *F58D5*_FW: 5’-TCCTGGATAATCCCCAAAAA-3’ | Eurofins Genomics | STNHN205 |
| *F58D5*_RV: 5’-CCCTGCCATTGATCTTGTTT-3’ | Eurofins Genomics | STNHN206 |
| *F54D10*_FW: 5’-TTGTGAGCTTATATCTCAGTTGTCG-3’ | Eurofins Genomics | STNHN207 |
| *F54D10*_RV: 5’-AGATTTGGTTAGAAATATCACCGC-3’ | Eurofins Genomics | STNHN208 |
| *Y38E10A*_FW: 5’-ACCGTTTAATAGGATTATTTGGG-3’ | Eurofins Genomics | STNHN209 |
| *Y38E10A*_RV: 5’-AAGTCTGCGGAATAATTGATGG-3’ | Eurofins Genomics | STNHN210 |
| *Y71H2B*_FW: 5’-GAGGAACCAAATCTGGCGTA-3’ | Eurofins Genomics | STNHN211 |
| *Y71H2B*_RV: 5’-TGAAAACTTGGAAAATCGGTG-3’ | Eurofins Genomics | STNHN212 |
| *Y41C4A*_FW: 5’-ATCAAGTTTCTGATTGCTCTTTCC-3’ | Eurofins Genomics | STNHN213 |
| *Y41C4A*_RV: 5’-AAAAACGTGATTTTTCAATTTTGC-3’ | Eurofins Genomics | STNHN214 |
| *Y38C1BA*_FW: 5’-CGCATAAATCCAACGTTCTCTG-3’ | Eurofins Genomics | STNHN215 |
| *Y38C1BA*_RV: 5’-AATCCATAAGTTTCGTGTTGGG-3’ | Eurofins Genomics | STNHN216 |
| *F49E11*_FW: 5’-GACACGACTTTAGAAACAACAGC-3’ | Eurofins Genomics | STNHN217 |
| *F49E11*_RV: 5’-TGGTATGGAGTCCCTATTTTGG-3’ | Eurofins Genomics | STNHN218 |
| *C24B9*_FW: 5’-TCATCTGTTATTTCGTCTCTTGC-3’ | Eurofins Genomics | STNHN219 |
| *C24B9*_RV: 5’-CGGTAATAATATGCTTTGTGGG-3’ | Eurofins Genomics | STNHN220 |
| *Y6G8*_FW: 5’-CATTCATTTCACCTGTTGGTTG-3’ | Eurofins Genomics | STNHN221 |
| *Y6G8*_RV: 5’-TCGGGAAGATAATCAAAATTCG-3’ | Eurofins Genomics | STNHN222 |
| *ZK470*_FW: 5’-CCAAAACGGCCAAGTATCAG-3’ | Eurofins Genomics | STNHN223 |
| *ZK470*_RV: 5’-TTGCACTCTTTCTCCTTCCG-3’ | Eurofins Genomics | STNHN224 |
| *T24C2*_FW: 5’-TTAAAACCATACAATTCTTCTCAGC-3’ | Eurofins Genomics | STNHN225 |
| *T24C2*_RV: 5’-GAATTCCCAATCAACAGAGAGC-3’ | Eurofins Genomics | STNHN226 |
| *him-8*_FW: 5’-ACAGAATGAATTCGCCGGTG-3’ | Eurofins Genomics | STNHN326 |
| *him-8*_RV: 5’-ACGCTTTAGGCGCCGAATTT-3’ | Eurofins Genomics | STNHN327 |
| *F56C11*_FW: 5’-ATGCCAGTGATAAGGAACGG-3’ | Eurofins Genomics | STNHN499 |
| *F56C11*_RV: 5’-TCACATCCCTTGTCGATGAA-3’ | Eurofins Genomics | STNHN500 |
| *Y71G12A*_FW: 5’-TCGAAATCAGGGAAAAATTGA-3’ | Eurofins Genomics | STNHN501 |
| *Y71G12A*_RV: 5’-ACGATTTTCGGGGAGTTTTT-3’ | Eurofins Genomics | STNHN502 |
| *D1007*_FW: 5’-AAAATATCAGGAAAGAGTTTCGG-3’ | Eurofins Genomics | STNHN503 |
| *D1007*_RV: 5’-TTTAAAGATTAAGGGTGGAGCG-3’ | Eurofins Genomics | STNHN504 |
| *B0205*_FW: 5’-ATCTGGCACCAAATATGAGTCG-3’ | Eurofins Genomics | STNHN505 |
| *B0205*_RV: 5’-AATCTCGATTTTCAAGGAGTGG-3’ | Eurofins Genomics | STNHN506 |
| *cewivar00073402*_FW: 5’-TCGAGGGTGTCAGGTTTTC A-3’ | Eurofins Genomics | STNHN511 |
| *cewivar00073402*_RV: 5’-CGAAGAGGAAAGAATGCGG G-3’ | Eurofins Genomics | STNHN512 |
| Alt-R CRISPR-Cas9 tracrRNA | IDT | Cat# 1072532 |
| *nsun-2*_crRNA: 5’-CCACUUCUUUUGGCUCCAACGUU UUAGAGCUAUGCU-3’ | IDT | N/A |
| *nsun-2*_repair DNA: 5’-CGACAGGAGGCGGTTTCAATG ATTCCACCAGAATTCTGACTTCTTTTGGCTCCAACTTCTGAGCATTTT-3’ | Eurofins Genomics | STNHN519 |
| *nsun-2*_FW: 5’-GTCCGAAGTCATCCGATCCT-3’ | Eurofins Genomics | STNHN520 |
| *nsun-2*_RV: 5’-GTTGTGTGGTCTTGCTTCCC-3’ | Eurofins Genomics | STNHN521 |
| *F49H12*_FW: 5’-ATATGTGAGTTTACCATCACTGGG-3’ | Eurofins Genomics | STNHN733 |
| *C46F4*_FW: 5’-AAGTGTTCAATGATTTTGTCTAATTG-3’ | Eurofins Genomics | STNHN735 |
| *F49H12*_RV: 5’-ACGTTTTGAAAAATTTGGTTGC-3’ | Eurofins Genomics | STNHN741 |
| *C46F4*_RV: 5’-TGACAGGAGAATACTTTTGAAGG-3’ | Eurofins Genomics | STNHN742 |
| Software/algorithm | Source | Identifier |
| Similarity Ensemble Approach (SEA) | https://sea.bkslab.org/ | RRID:SCR_023754 |
| MEGA11 | https://www.megasoftware.net/ | RRID:SCR_000667 |
| AutoDock Vina 1.2.5 | https://vina.scripps.edu/ | RRID:SCR_011958 |
| Protein-Ligand Interaction Profiler (PLIP) | https://plip-tool.biotec.tu -dresden.de/plip-web/ plip/index | RRID:SCR_024173 |

N/A, not applicable; RRID, research resource identifier (https://www.rrids.org/).
